# Malaria reduction drives childhood stunting decline in Uganda: a mixed-methods country case study

**DOI:** 10.1093/ajcn/nqac038

**Published:** 2022-02-14

**Authors:** Emily C Keats, Richard B Kajjura, Anushka Ataullahjan, Muhammad Islam, Breagh Cheng, Ahalya Somaskandan, Kimberly D Charbonneau, Erica Confreda, Rachel Jardine, Christina Oh, Peter Waiswa, Zulfiqar A Bhutta

**Affiliations:** Centre for Global Child Health, Hospital for Sick Children, Toronto, Ontario, Canada; Makerere University School of Public Health, Kampala, Uganda; Centre for Global Child Health, Hospital for Sick Children, Toronto, Ontario, Canada; Centre for Global Child Health, Hospital for Sick Children, Toronto, Ontario, Canada; Centre for Global Child Health, Hospital for Sick Children, Toronto, Ontario, Canada; Centre for Global Child Health, Hospital for Sick Children, Toronto, Ontario, Canada; Centre for Global Child Health, Hospital for Sick Children, Toronto, Ontario, Canada; Centre for Global Child Health, Hospital for Sick Children, Toronto, Ontario, Canada; Centre for Global Child Health, Hospital for Sick Children, Toronto, Ontario, Canada; Centre for Global Child Health, Hospital for Sick Children, Toronto, Ontario, Canada; Makerere University School of Public Health, Kampala, Uganda; Centre for Global Child Health, Hospital for Sick Children, Toronto, Ontario, Canada; Dalla Lana School of Public Health, University of Toronto, Toronto, Ontario, Canada; Centre of Excellence in Women and Child Health, the Aga Khan University, Karachi, Pakistan

**Keywords:** child, infant, nutrition, height, length, linear growth, stunting, malaria, Uganda

## Abstract

**Background:**

Uganda has achieved a considerable reduction in childhood stunting over the past 2 decades, although accelerated action will be needed to achieve 2030 targets.

**Objectives:**

This study assessed the national, community, household, and individual-level drivers of stunting decline since 2000, along with direct and indirect nutrition policies and programs that have contributed to nutrition change in Uganda.

**Methods:**

This mixed-methods study used 4 different approaches to determine the drivers of stunting change over time: *1*) a scoping literature review; *2*) quantitative data analyses, including Oaxaca–Blinder decomposition and difference-in-difference multivariable hierarchical modeling; *3*) national- and community-level qualitative data collection and analysis; and *4*) analysis of key direct and indirect nutrition policies, programs, and initiatives.

**Results:**

Stunting prevalence declined by 14% points from 2000 to 2016, although geographical, wealth, urban/rural, and education-based inequalities persist. Child growth curves demonstrated substantial improvements in child height-for-age *z*-scores (HAZs) at birth, reflecting improved maternal nutrition and intrauterine growth. The decomposition analysis explained 82% of HAZ change, with increased coverage of insecticide-treated mosquito nets (ITNs; 35%), better maternal nutrition (19%), improved maternal education (14%), and improved maternal and newborn healthcare (11%) being the most critical factors. The qualitative analysis supported these findings, and also pointed to wealth, women's empowerment, cultural norms, water and sanitation, dietary intake/diversity, and reduced childhood illness as important. The 2011 Uganda Nutrition Action Plan was an essential multisectoral strategy that shifted nutrition out of health and mainstreamed it across related sectors.

**Conclusions:**

Uganda's success in stunting reduction was multifactorial, but driven largely through indirect nutrition strategies delivered outside of health. To further improve stunting, it will be critical to prioritize malaria-control strategies, including ITN distribution campaigns and prevention/treatment approaches for mothers and children, and deliberately target the poor, least educated, and rural populations along with high-burden districts.

## Introduction

Stunting is the product of poor nutrition in utero and in early childhood (1, 2). Children who are stunted have a height-for-age *z*-score (HAZ) >2 SDs below the population median, and have higher rates of mortality, morbidity, and suboptimal development that affects outcomes in adulthood and in subsequent generations (1, 2). Uganda, a landlocked country in East-Central Africa with a population >42 million (3, 4), has achieved considerable reduction in stunting over the past 20 y (5), earning it the title of an Exemplar Nation (6). However, the country's population is growing at a rate of 3.2%, which can cause a significant strain on resources (7).

National under-5 stunting prevalence declined from 41% in 2000 to 27% in 2016 (8–11), although improvements have not been uniform. Parts of Western and Northern Uganda have lagged behind due to differing contexts related to sociodemographic, agricultural, climate, conflict, cultural (e.g., feeding practices), and service delivery factors. In addition, the violent Lord's Resistance Army movement resulted in internal displacement, chronic food insecurity, and poor access to healthcare (12–14). Uganda has also experienced a rise in severe weather events (15), like droughts and floods, which cause significant damage to crops, livelihoods, and even infrastructure, dramatically affecting food and nutrition security levels, with evidence of the link between climate change and linear growth (16).

Conversely, there has been substantial progress in several key areas that have directly and indirectly impacted health and nutrition. For example, gross domestic product per capita rose by 65.6%, from $1252 in 2000 to $2073 in 2016 (17), alongside a 45% reduction in poverty (18). The proportion of women with no education decreased from 21.9% in 2000 to 9.6% in 2016, and the total fertility rate reduced from 6.9 births per woman to 5.4 (8, 11). Importantly, from 2009 to 2014 the percentage of households with ≥1 insecticide-treated mosquito net (ITN) or long-lasting insecticidal net increased to >90% (19, 20), and over three-quarters of children aged <5 y were sleeping under bed nets (19, 20).

Malaria prevention and treatment play a critical role in improving maternal and child health, and Uganda is considered one of the highest malaria burden countries in the world (21, 22). Risks are compounded by climate change, which can increase transmission and infection rates (23, 24). Malaria infection during pregnancy has been linked to numerous adverse outcomes, including stillbirth, low birthweight, neonatal mortality, and maternal anemia (25, 26). ITN use and intermittent preventative treatment during pregnancy (IPTp), along with therapeutic approaches in childhood, are therefore critical to achieving sustainable health gains in the Ugandan context. Uganda's improvement in these and other key areas could link to a multifactorial stunting reduction success story. Therefore, the objective of this study was to conduct a systematic and in-depth assessment of the drivers of stunting reduction in Uganda from 2000 to 2016. Specifically, this study aimed to: *1*) quantitatively examine the determinants of stunting reduction and decompose long-term stunting change into relative contribution from key drivers; *2*) explore national- and community-level perspectives on stunting progress; and *3*) generate a systematic landscape of major nutrition-relevant policies and programs that have contributed to progress.

## Methods

### Study design

This study involved 4 methodological approaches of inquiry: a literature review, retrospective quantitative data analysis, qualitative data collection and analysis, and a policy and program review. The conceptual framework was adapted from the 2013 Lancet Nutrition Series, which outlines the basic, underlying, and immediate causes of child stunting (1) (**Supplemental Figure 1**). This framework informed the study design and data analyses. Ethics approval was obtained from the Makerere University School of Public Health Higher Degrees Research Ethical Committee (Uganda), the Uganda National Council of Science and Technology, and the Research Ethics Board at the Hospital for Sick Children (Canada).

### Scoping review

A systematic search of peer-reviewed and gray literature was conducted to synthesize information on factors that might have contributed to a reduction in stunting in Uganda. Searches were conducted in 13 online databases and 16 gray literature sources. Multiple search terms relating to “stunting,” “child,” and “Uganda” were used. Records were included if they were published in 1990 or later, included an under-5 population in Uganda, and examined ≥1 determinant of chronic undernutrition or a reduction in stunting. Data were extracted by a single reviewer for 56 studies that were included in our analysis (**Supplemental Table 1, Supplemental Figure 2**).

### Quantitative methods

Uganda Demographic and Health Surveys (UDHS) (2000, 2006, 2011, 2016) were the primary datasets used in this study. These nationally representative household surveys use comparable, standardized methods with data for a wide range of indicators relating to population demographics, health, and nutrition. Sample sizes for each survey year based on available anthropometry data for index (youngest) children aged <5 y are presented in **Supplemental Table 2**. A detailed quantitative methodology can be found elsewhere (6).

### Outcomes and covariables

The main outcomes examined were HAZ and stunting prevalence (HAZ >2 SDs below the population median), which was calculated using WHO child growth standards (27). Covariables that aligned with Supplemental Figure 1 and were available within UDHS were selected and analyzed as distal, intermediate, and proximal predictors of HAZ change over time.

### Statistical analysis

Equity analyses were performed using standardized and well-established methods to examine stunting prevalence by region, wealth, maternal education, urban compared with rural residence, and child sex. Wealth quintiles were obtained using principal components analysis of household asset data. Slope index of inequality (SII) and concentration index (CIX) were used to estimate absolute and relative socioeconomic inequalities, respectively. In addition, absolute and relative declines in stunting prevalence for each region were calculated using average annual percentage-point rate of change (AARC) in stunting and compound annual growth rates (CAGRs).

Kernel density plots were created to study population changes in child HAZ over time. Child growth curves (Victora curves) were plotted using smooth local polynomial regressions to examine the growth faltering process from birth to 59 mo of age (28). Piecewise linear splines were fitted to the polynomial curves, where knots indicate major changes in child growth. Two multivariable analyses were conducted using a series of stepwise linear mixed-effect regression models and hierarchical modeling. The 4 cross-sectional surveys used in this analysis were assembled into panel datasets, and difference-in-difference (DID) analyses were used with time × covariable interaction terms to estimate whether change in a proposed HAZ predicator led to change in HAZ over the study period. Regression-based Oaxaca–Blinder decomposition was also conducted to understand the major predictors and their relative contribution to stunting decline in Uganda. All analyses were conducted with Stata version 14.0 (StataCorp LLC) and accounted for survey design and weighting.

### Qualitative methods

Qualitative research was conducted between June and September 2020 using key informant interviews (KIIs) at the national and district levels and focus group discussions (FGDs) with mothers in communities. The KIIs were conducted with national stakeholders (*n* = 17) that had extensive experience in the design, implementation, or evaluation of direct and indirect nutrition interventions across sectors. Interviewers asked respondents to describe the drivers of and barriers to childhood stunting reduction. KIIs with district stakeholders and FGDs with mothers were conducted in Serere and Kasese districts. Serere is located in the Teso subregion, which has shown accelerated stunting progress, whereas Kasese is found in the Tooro subregion, which has lagged in stunting reduction. It should be noted that the regional qualitative inquiry was not intended to be representative of Uganda at large, rather it was meant to signal some of the key drivers of stunting reduction on a community level that could be relevant for other regions. Interviews with district stakeholders (*n* = 9 per district), selected based on their experience in delivering nutrition-related services, explored local nutrition trends, community practices, and the implementation of direct and indirect nutrition interventions. FGDs with mothers who gave birth to children during 2 time periods (1995–2000 and 2011–2016) sought to explore women's experiences and insights regarding pregnancy, breastfeeding, nutrition practices, and general trends in child nutrition. Specifically, 12 FGDs were conducted in total; 3 for each time period per district.

### Qualitative data analysis

The KII and FGD interview transcripts were audio-recorded and transcribed verbatim. The information was translated into English and entered into Atlas.ti software. Deductive analysis was performed using a data discharge matrix, based on the conceptual framework (Supplemental Figure 1). The analysis of the KII and FGD responses was performed by 2 coding persons simultaneously, allowing for the exchange of ideas on the responses obtained and the codes employed by the research team. The identified codes, concepts, and emerging themes were used to explore the drivers, facilitators, and barriers of stunting reduction. Relevant quotes were identified to support the themes that emerged during the analytic process.

### Policy and program review

Important direct and indirect nutrition laws, policies, programs, and initiatives since 1990 were gathered through an iterative approach. Related literature was systematically identified and compiled into a timeline to share with expert stakeholders to gain insight on relevance to nutrition in Uganda and any missing initiatives. Additional policy/program documents suggested by stakeholders were then added to the timeline in an iterative fashion, until a consensus was reached. The initiatives were later rated by in-country nutrition experts and research team members to reflect the relative importance of each to the observed childhood reduction in stunting. Available assessments/evaluations were taken into consideration.

## Results

**FIGURE 1:**
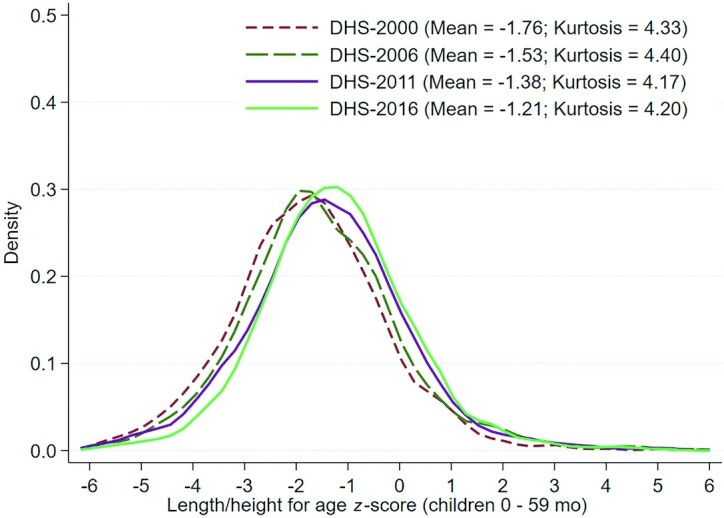
Kernel density plots representing population HAZ score distributions for 2000 (*n* = 5144), 2006 (*n* = 2367), 2011 (*n* = 2069), and 2016 (*n* = 4405) in children aged <5 y. DHS, Demographic and Health Survey; HAZ, height-for-age *z*-score.

Prevalence of childhood stunting in Uganda declined by 14% points over the study period (2000–2016). **Figure 1** shows the distribution of child HAZs for each survey year examined. The curve has shifted rightward over time from a mean HAZ of −1.76 in 2000 to −1.21 in 2016, underscoring nutritional gains for all under-5s. From 2000 to 2006, there was a narrowing of the curve (kurtosis: 4.33 to 4.40) indicating that more Ugandan children were clustering around the mean HAZ in 2006 or, in other words, inequalities improved. This trend reversed between 2006 and 2011, but became positive once again by 2016.

**FIGURE 2:**
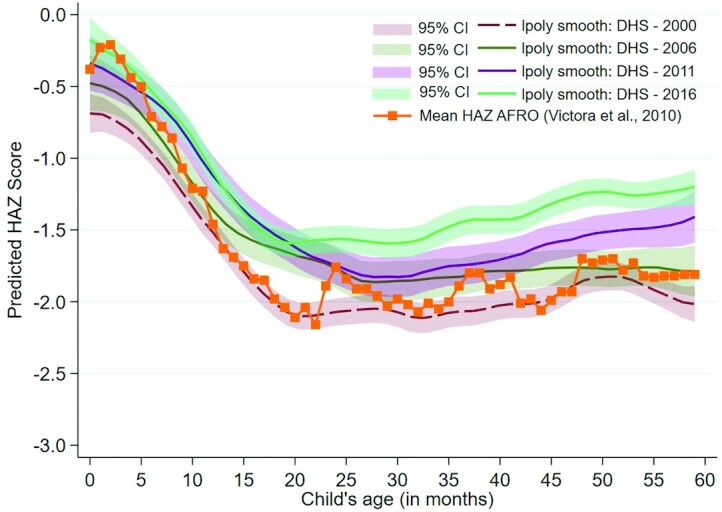
Victora curves for children aged <5 y for 2000 (*n* = 5144), 2006 (*n* = 2367), 2011 (*n* = 2069), and 2016 (*n* = 4405), including mean HAZ scores for Africa (HAZ AFRO) in 2010 (28). AFRO, World Health Organization Regional Office for Africa; DHS, Demographic and Health Survey; HAZ, height-for-age *z*-score.

**Figure 2** presents child HAZ plotted against child age, enabling the examination of changes in the growth faltering pattern. Mean HAZ scores from the WHO African region included in Victora's global analysis are added for comparison (28). Improvements in Ugandan children's HAZ trajectory can be observed over the 16-y study period, because the entire curve has shifted upwards over time. The 2000 curve intercepts at the lowest point of all years studied (HAZ = −0.60) whereas the 2016 intercept reaches the highest point (HAZ = −0.14), indicating improvements in HAZ at birth that reflect better maternal nutrition and maternal and newborn care practices over the study period. HAZ declines between 0 and 6 mo, and slopes appear similar across curves suggesting sustained inadequate breastfeeding practices. Growth faltering continues post 6 mo, although the curve is flatter in 2016 than in previous years suggesting improved complementary feeding practices and diet diversity, home environments, and/or care-seeking for illness. The period of decline in 2016 is the shortest of all years examined, stopping at ∼17 mo. From this point, HAZ begins a steady recovery that continues through to the 60-mo mark. From 30 mo onwards, the 2016 HAZ scores remained well above those of 2011, highlighting that children in 2016 are significantly taller and healthier than in previous years. **Supplemental Figure 3**A–E shows Victora curves with added splines, which detail inflection points where the slope of the HAZ curve has changed.

**FIGURE 3:**
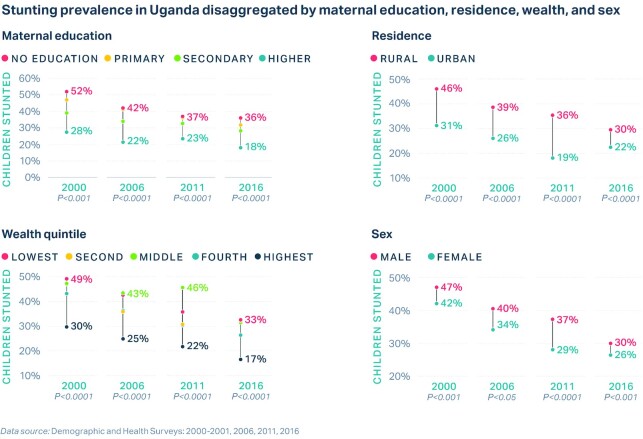
National stunting change in children aged <5 y over time (2000 to 2016) disaggregated by maternal education (*n* = 5047), urban compared with rural residence (*n* = 5047), wealth quintile (*n* = 5047), and child sex (*n* = 5047).

Uganda experienced some variation in stunting decline by region (**Supplemental Figure 4**A). The Western region demonstrated the largest absolute reduction of 1.2% per annum, whereas the Northern region experienced the slowest absolute change (AARC = 0.8%) (Supplemental Figure 4B). In terms of relative change, Eastern Uganda performed the best (CAGR = 3.5%), whereas the Northern region demonstrated the smallest relative reduction (CAGR = 2.2%) (Supplemental Figure 4B). Analysis by district (2016) revealed substantial differences in stunting prevalence, even within a given region (**Supplemental Figure 5**). Differences also exist by maternal education, residence, wealth, and child sex (**Figure 3**), whereby children of educated mothers and urban, rich, girl children had a lower risk of being stunted. However, disparities declined over the study period, particularly by residence and wealth, driven by greater improvements among rural children and those in the poorest wealth quintiles (Figure 3). It should be noted that advanced equity analyses (SII, CIX) demonstrated increasing socioeconomic inequalities between 2011 and 2016 (**Supplemental Figure 6**A-B).

**FIGURE 4:**
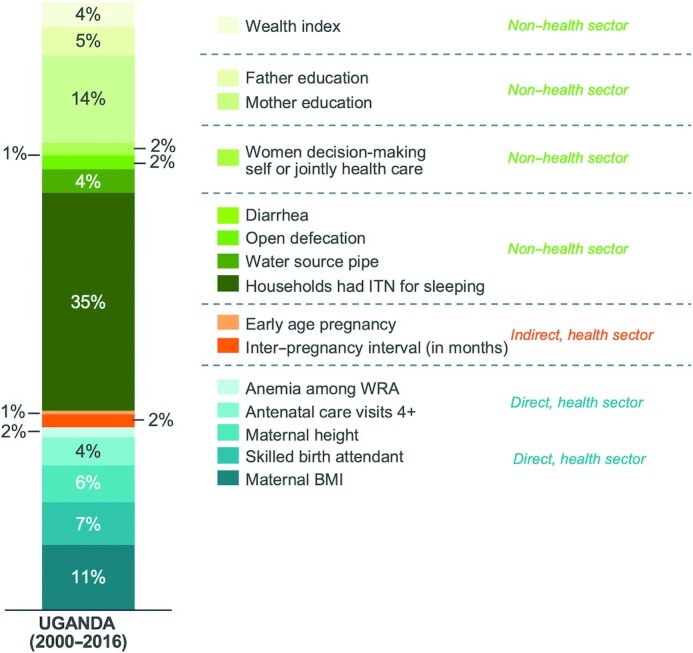
Decomposing predicted change in HAZ, with relative contribution of determinants (%), in children aged <5 y from 2000 to 2016 (*n* = 5098). Statistical analysis: Oaxaca–Blinder decomposition. HAZ, height-for-age *z*-score; ITN, insecticide-treated bed net; WRA, women of reproductive age.

The decomposition results identified 4 main explanatory factors for predicted HAZ change in children aged <5 y between 2000 and 2016 (**Supplemental Table 3**, **Figure 4**). The primary explanatory factor was increased coverage of ITNs (35%), followed by better maternal nutrition (19%)—defined as anemia prevalence, height, and BMI—improved maternal education (14%), and improved maternal and newborn healthcare (11%)—defined as antenatal care and skilled birth assistance. Together, these 4 factors accounted for nearly 80% of the change in HAZ over the study period. Other important factors included improved paternal education (5%), greater wealth (4%), access to clean water (4%), improved women's empowerment (2%), reduced open defecation (2%), longer interpregnancy intervals (2%), reduced diarrhea (1%), and fewer adolescent births (1%). Overall, the decomposition explained 82% of HAZ change (0.45 SDs).

The key explanatory factors remained consistent when decomposition was undertaken for the <6 mo-, 6–23 mo-, and ≥24-mo children, with ITN coverage being the primary driver of HAZ change across age groups (**Supplemental Tables 4–6**). The results of the DID analysis (**Supplemental Table 7**) showed that improved wealth, paternal education, ITN ownership, and acute respiratory infection incidence were associated with HAZ change in children aged <5 y between 2000 and 2016.

National stakeholders and mothers from Teso and Tooro districts participated in KIIs (16) and FGDs (13), respectively. Emergent themes and supporting quotes are presented in **Supplemental Table 8**.

The qualitative findings support the decomposition analysis, with national stakeholders noting both an observed reduction in malaria and its link with child growth and development. Many attributed this reduction to the increased coverage of mosquito nets, but also to better health coverage, which improved the availability of quality medicines (i.e., artemisinin-based combination therapy) to treat malaria. The FGD findings with mothers also supported these observations, with mothers of children born earlier describing high rates of malaria in contrast to mothers of children born later, who spoke of reduced rates. Women's education was a key determinant of stunting reduction that was also highlighted through the qualitative findings, along with the importance of women's empowerment as a means of engaging in better health practices for both mothers and their children. Mothers in Teso especially emphasized that empowered women had improved access to resources, including health resources, better decision-making ability, and improved dietary diversity; they were in a position to refute food taboos and other harmful practices. Underlying women's empowerment was the ability to control finances and reduce reliance on men, which allowed women to make decisions regarding their own and their children's welfare. Other drivers, such as improved wealth, access to clean and safe water and sanitation facilities, and reductions in childhood illness, were also noted, although adolescent pregnancy was still cited as a major concern despite the reduction in adolescent fertility from 172 to 121 births per 1000 girls.

The qualitative findings also highlighted regional differences in stunting reduction drivers including conflict, poverty, urbanization, diet diversity, and cultural norms (Supplemental Table 7). For example, feeding practices, including child dietary diversity scores, were better in Teso than in Tooro region. The availability of diverse nutritious foods, proper food preparation, and appropriate feeding practices were highlighted by respondents as contributing to improved nutrition of children in Teso. Local fresh lakes allowed for easily accessible fresh fish, and farming of millet, pea nuts, cassava, simsim, and cattle were common. The cow farming in Teso region allowed for greater household access to animal-source protein. This was further enhanced by lower poverty and improved maternal education over time, which could have prompted more informed and better food choices, including diverse nutritious foods. On the other hand, dietary customs and availability, including a preference for less-nutritious, carbohydrate-based staple foods (e.g., matooke and potatoes) and reduced plant and animal-rich protein foods, were described as a barrier to improving dietary intake in Tooro region.

**FIGURE 5:**
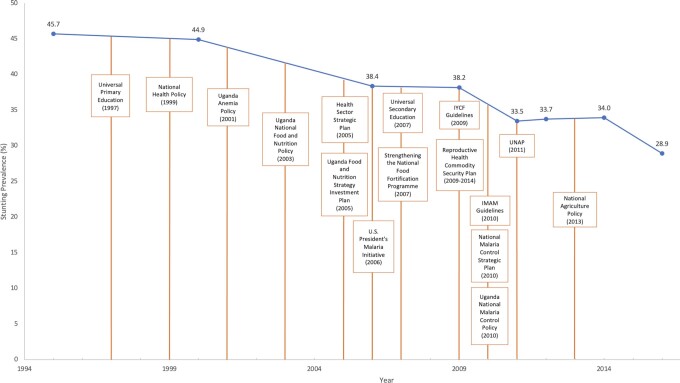
Key direct and indirect nutrition policies and programs and stunting prevalence in children aged <5 y (%) decline in Uganda. IMAM, integrated management of acute malnutrition; IYCF, infant and young child feeding; UNAP, Uganda Nutrition Action Plan.

The policy and program review found 69 nutrition-relevant initiatives; just over half were identified as important or very important to stunting decline in Uganda. Many of these critical initiatives were implemented 2–5 y before inflection points in the stunting reduction curve (**Figure 5**), highlighting their slight lag in effect on nutrition. Aligned with the findings of the multivariable analyses, the US President's Malaria Initiative (2006), the National Malaria Control Strategic Plan (2010–2015), and the Uganda National Malaria Control Policy (2010–2015) were instrumental initiatives that focused on the prevention and management of malaria, including the successful delivery of ITNs to households. The Education Sector Strategic Plan (2004–2015) and the Universal Secondary Education program (2007) were important for increasing girls’ school attendance and having downstream, positive effects on women's empowerment, employment opportunities, adolescent births, and the intergenerational transfer of malnutrition. The Uganda Anemia Policy (2003) and the National Food Fortification Programme (2007) improved women's micronutrient status, although pathways to better maternal nutrition can also exist through increases in education, empowerment, and greater access and use of health services. Continued strengthening of Uganda's health system through outreach by Village Health Teams, community-level behavior-change-communication strategies, and an increased number of health facilities improved access to quality health services. In addition, the Integrated Case Management of Childhood Illnesses (iCCM) was believed to underlie noted improvements in childhood stunting.

The 2011 Uganda Nutrition Action Plan (UNAP) was consequential in mainstreaming nutrition beyond health, across Ministries, Departments, and Agencies (MDA). The qualitative findings from the national and regional respondents identified UNAP as an important platform for the coordination of nutrition-related activities across MDA, and described political commitment to improving nutrition as reflected in the implementation of multisectoral policies and programs. Despite political commitment, study participants highlighted that financial support often limited program scale-up, and interagency collaboration was a problem. Other key initiatives targeted maternal and child health and nutrition, water sanitation and hygiene (WASH), and poverty reduction, overlapping with the findings of the decomposition analysis. Expanded policy and program findings can be found in **Supplemental Table 9**.

## Discussion

Uganda's success in reducing childhood stunting was the summation of political will and investments in nutrition actions that spanned several MDA. Based on the decomposition, 70% of the explained change in HAZ was the result of indirect nutrition strategies (30% from direct interventions), and 67% could be attributed to strategies delivered outside of the health sector (33% within health). Although most malaria-related interventions would be delivered within the health sector, ITNs are classified as nonhealth because they are mainly delivered through mass distribution campaigns that are not coordinated through the health system. This study has demonstrated that, despite some reductions in inequalities, disparities in stunting prevalence remain that relate to geography, socioeconomic characteristics, access to nutritious foods, and community-/individual-level factors such as cultural norms. To further accelerate stunting reduction in the coming decade and to reach Uganda's targets, these gaps will need to be addressed through specific targeting of vulnerable groups.

The multisectoral stunting reduction story in Uganda has been documented in other Exemplar countries such as Ethiopia, Nepal, and Peru (29), although each exhibited a slightly different pathway to success based on country context and status of determinants, meaning that there is no single silver bullet. For example, success in Ethiopia depended on agricultural-related inputs, and subsequently, food security improvements, whereas in Peru, population migration from mountainous regions to urban areas was a key driver. As was the case in other Exemplar nations that had a starting stunting prevalence of ∼40%, focusing on women's and mothers’ health and nutrition broadly appears to be a critical component that addresses the intergenerational transfer of stunting. Girls’ education and improved fertility indicators are also strong contributors of change (29), underscoring the overarching importance of female empowerment, which holds true in the Ugandan context. Similar to the work of the International Food Policy Research Institute, where bed net coverage accounted for 72% of the explained change in HAZ in Zambia (30), the Uganda case study has confirmed the important, and novel, link between antimalaria interventions and child stunting. Although our decomposition analysis includes ITN use alone, it is important to note that overall health system strengthening in Uganda allowed for other malaria-reduction interventions, including early identification and community-based testing, treatment, and referral, which could have reduced case counts over time. Although it is difficult to directly compare malaria change over time because of known spatiotemporal trends (31) in incidence and the impact of climate-related events on transmission (23), the dramatic improvement in regions with a very high malaria burden across Northern and Eastern Uganda between 2000 and 2015 (32) aligns with districts of low stunting prevalence in 2016 (**Supplemental Figure 7**). One would need to consider the slight lag in effect of maternal malaria reduction on childhood stunting when using UDHS data (i.e., the increase in malaria seen in 2016 would not be reflected in 2016 stunting prevalence). Examining other malaria indicators (e.g., household ownership of ITNs and children sleeping under mosquito nets) against stunting prevalence also reveals a negative trend (**Supplemental Figure 8**A-B) (11). However, this is not always the case; for example, Teso has traditionally recorded high malaria cases (52% prevalence in 2016) (11), and our qualitative findings have pointed to other factors that have contributed to the reduced stunting prevalence in Teso specifically.

The pathway of effect of ITN use on stunting is likely through reduced maternal malarial burden, reduced intrauterine growth restriction (IUGR), and improved HAZ at birth—a finding that was mirrored in the Victora curve analysis. The link between malaria during pregnancy, IUGR, and adverse birth outcomes has been well established (33–37). In Uganda, associations have also been shown between malaria in pregnancy and increased risk of malaria and other infections in infancy (21). Placental malaria is one of the primary mechanisms through which IUGR takes place, because it leads to impaired nutrient transport to the fetus. Importantly, a recent individual participant data analysis demonstrated that risk of small-for-gestational age was high in pregnant women infected with *Plasmodium falciparum*, despite treatment with highly effective drugs (38), underscoring the critical nature of malaria prevention through interventions such as ITNs or indoor residual spraying (IRS). A second pathway of effect could be through reduced parasitemia in children and lower illness events per child per year. Uganda's success in addressing its malaria burden lay in a coordinated effort that combined the scale-up of such preventive efforts with management-based approaches, including iCCM and an increase in drug shops. These gains were achieved against the backdrop of negative climate change effects on growth and malarial burden. Not only did ITN ownership increase from 13% in 2000 to a high of 90% in 2014, mostly as a result of mass distribution campaigns, but IPTp (1 dose) reached 77% of pregnant women, and 63% of children took antimalarials for fever in 2016 (11). The 2021 WHO consolidated guidelines for malaria continue to recommend vector control through ITNs and IRS, along with preventive chemotherapies and mass drug administration for vulnerable populations, and case management (39). Given the recent propensity for malaria mutations that confer drug resistance in East Africa (40), preventive efforts will be paramount.

The importance of malaria reduction in mothers and children links to anemia, which can be caused by malarial and other infections, iron deficiency, and hemoglobinopathies. The decomposition analysis also found a 2% contribution to HAZ change through reductions in anemia in women of reproductive age—a finding that could also link to improvements in low birthweight (41). Uganda has been highlighted as an Exemplar in anemia prevalence reduction as well (42% in 2006, falling to 32% in 2016) (9, 11), and forthcoming analyses will attempt to understand the complex drivers of this reduction over time. Although direct nutrition actions to improve mothers’ and children's health, including iron supplementation, are clearly critical, this study highlighted the prominence of indirect actions for driving stunting change in Uganda. Poverty reduction, educational initiatives, improved access to WASH, reductions in adolescent births and improved birth spacing, and increased women's decision-making together contributed to the national-level childhood decline in stunting. However, qualitative findings have underscored the differences in these determinants that exist at the district, community, and household levels and provide a template for policy and programmatic action that will ensure the most vulnerable are not left behind. A district-level assessment of the factors outlined within this study would assist with such a targeting strategy.

This study was not without limitations. Firstly, we relied on UDHS data, which provide a cross-sectional snapshot of determinants over time and therefore cannot assume causality. However, using panel UDHS surveys and examining variable interactions with time through the DID analysis has allowed insight into effect modifiers of the time-HAZ relationship. Secondly, quantitative data availability for certain key determinants, such as conflict severity, climate change, agricultural productivity, food security, dietary intake, and birthweight, were lacking. The unexplained portion of the decomposition analysis (18%) could therefore reflect some of these factors, many of which were highlighted through the qualitative research. In addition, there are no maternal malarial prevalence data for Uganda, hence we were unable to confirm the reduction in prevalence over time. Thirdly, there are limitations to the decomposition approach, which have been previously outlined in the literature (30, 42, 43). Lastly, the qualitative data collection focused on 2 key districts that represented progress and lack of progress in stunting reduction, but cannot be interpreted as being nationally representative.

Despite Uganda's success over the past 2 decades, national stunting prevalence remains high at 29%, so strategies that deliberately target the poor, least educated, and rural populations, along with high-burden districts, are required. The agricultural sector has been a major driver of poverty reduction through employment, and many Ugandans rely on subsistence farming for household consumption. Because of this, national food security has been relatively high over time, although foods consumed are not always diverse, which largely depends on regional crop varieties and education levels coupled with household wealth. However, national investments in new technologies to improve agricultural productivity have been low, so the sector is stalling as land is used up and processes remain inefficient. Coupled with this, Uganda is prone to climate shocks, which contribute to low productivity and food insecurity. Developing expanded social protection schemes for vulnerable populations to manage the increasing impact of climate change and investing in new technologies and nutrition-sensitive innovations in the agriculture sector will be critical. The findings of this study have underscored the importance of efforts that are multisectoral, and support Uganda's evolution of UNAP. In July 2019, the Office of the Prime Minister and other key stakeholders developed the strategic direction, implementation framework, and monitoring and evaluation plan for the second UNAP (UNAP II), which is currently being implemented in select districts (44). Overcoming some of the challenges of UNAP I, UNAP II aims to enable further sustainable cross-sectoral nutrition integration efforts through program-based budgeting and implementation at the local government levels.

In conclusion, as our study suggests, Uganda has seen a remarkable reduction in childhood stunting rates through maternal and child survival strategies, including malaria reduction, and nutrition interventions. However, stunting rates remain high, and reduction can be further accelerated by focusing on integrated strategies that include multisectoral direct and indirect nutrition interventions. These are likely to have positive effects on other forms of malnutrition too, including wasting and concurrent stunting and wasting, which also demonstrate regional variability in Uganda. Along with increasing family planning uptake, ensuring that adolescent-friendly health services are available, and keeping girls in school, this must include the continued prioritization of malaria-reduction strategies. These strategies include ITN distribution campaigns, mass indoor spraying, and prevention/treatment for mothers and children through health system strengthening that includes community-based approaches. Promoting antenatal and postnatal care for mothers, including education around breastfeeding and complementary feeding practices through community-based initiatives, are other no-regret actions. Lastly, successful implementation of UNAP II and the harmonization of national-level coordination and local government-based programming for nutrition will be important to maintain the focus and commitment to nutrition needed, particularly in the post-COVID era.

## Supplementary Material

nqac038_Supplemental_File

## Data Availability

Quantitative data described in the manuscript, code book, and analytic code are publicly and freely available without restriction from dhsprogram.com. Qualitative data described in the manuscript will not be made available because of ethical considerations around participant anonymity.

## Notes

This study was funded by 2 grants to the Centre for Global Child Health, Hospital for Sick Children from Gates Ventures and the Bill & Melinda Gates Foundation (grant number: INV-002458). The funders had no role in the design of the study, or in the analysis or interpretation of the data.

Supplemental Figures 1–6 and Supplemental Tables 1–9 are available from the “Supplementary data” link in the online posting of the article and from the same link in the online table of contents at https://academic.oup.com/ajcn/.

ECK and RBK are joint first authors.

Abbreviations used: AARC, average annual rate of change; CAGR, compound annual growth rate; DID, difference-in-difference; FGD, focus group discussion; HAZ, height-for-age *z*-score; iCCM, Integrated Case Management of Childhood Illnesses; IPTp, intermittent preventative treatment during pregnancy; IRS, indoor residual spraying; ITN, insecticide-treated net; IUGR, intrauterine growth restriction; KII, key informant interview; MDA, ministries, departments, and agencies; UDHS, Uganda Demographic and Health Survey; UNAP, Uganda National Action Plan; WASH, water, sanitation, and hygiene.
