# Supplementary material for: Malaria reduction drives childhood stunting decline in Uganda: a mixed-methods country case study
Source: Am J Clin Nutr. 2022 Feb 14;115(6):1559–68. doi: 10.1093/ajcn/nqac038 (PMC9170463; doi:10.1093/ajcn/nqac038)
Supplement: nqac038_Supplemental_File [file nqac038_supplemental_file.docx]

## **Supplementary Figure 1:** Conceptual framework showing distal, intermediate and proximal determinants of stunting.


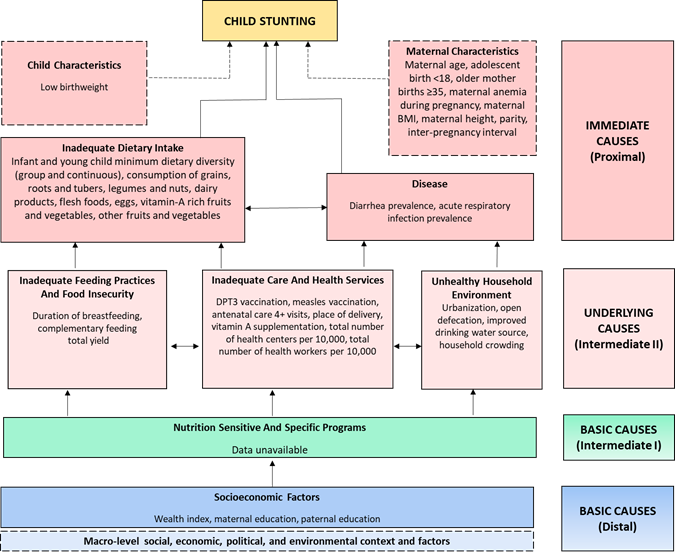


## **Supplementary Figure 2:** Flow diagram for study selection.


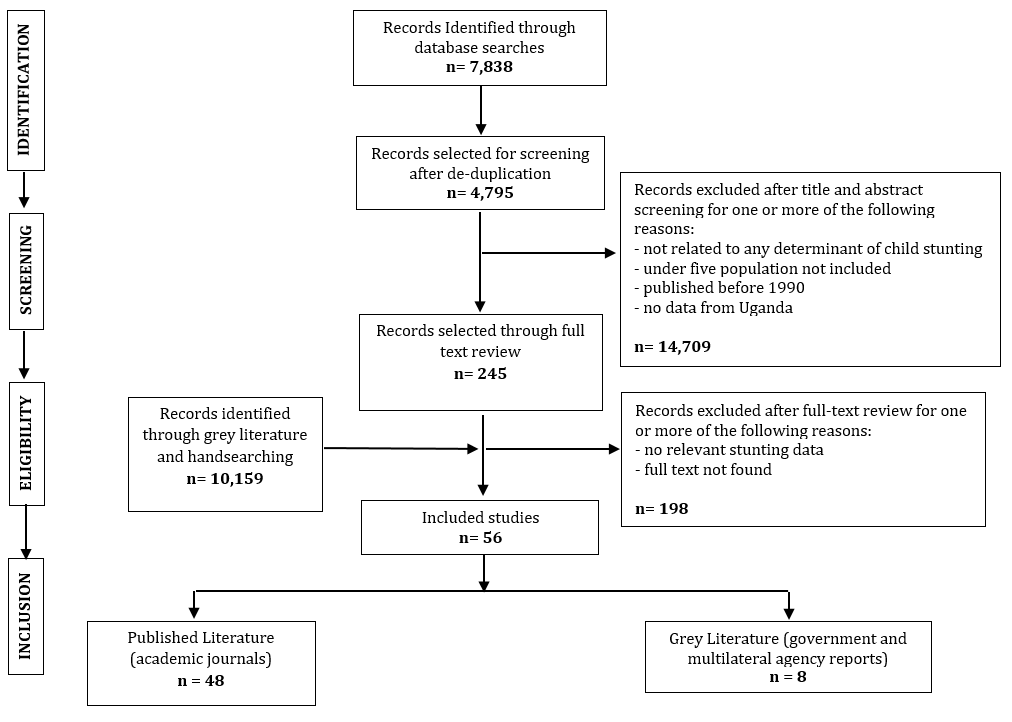


## **Supplementary Table 1:** Reference list of studies reviewed.

| **No** | **Reference** |
| --- | --- |
| 1 | Nshakira-Rukundo E, Mussa EC, Gerber N, *et al.* Impact of Community-Based Health Insurance on Child Health Outcomes: Evidence on Stunting from Rural Uganda. *Cent Dev Res Pap Dev Policy* 2019;:1–39.https://ageconsearch.umn.edu/record/281279/files/ZEF_DP_269.pdf |
| 2 | Biondi D, Kipp W, Jhangri GS, *et al.* Risk Factors and Trends in Childhood Stunting in a District in Western Uganda. *J Trop Pediatr* 2011;**57**:24–33. doi:10.1093/tropej/fmq043 |
| 3 | Vella V, Tomkins A, Nviku J, *et al.* Determinants of Nutritional Status in South-west Uganda. *J Trop Pediatr* 1995;**41**:89–98. doi:10.1093/tropej/41.2.89 |
| 4 | Ahn N, Shariff A. Determinants of child height in Uganda: A consideration of the selection bias caused by child mortality. *Food Nutr Bull* 1995;**16**:49–59.https://www.scopus.com/inward/record.uri?eid=2-s2.0-0029480792&partnerID=40&md5=3b49f513dce9e4592667a49e08ec4d0a |
| 5 | Kim H. In the wake of conflict: the long-term effect on child nutrition in Uganda. *Oxford Dev Stud* 2019;**47**:336–55. doi:10.1080/13600818.2019.1578877 |
| 6 | Lawson D, Appleton S. Child health in Uganda: Policy Determinants and Measurement. *Eur J Dev Res* 2007;**19**:210–33. doi:10.1080/09578810701289022 |
| 7 | Yang Y, Kaddu G, Ngendahimana D, *et al.* Trends and determinants of stunting among under-5s: evidence from the 1995, 2001, 2006 and 2011 Uganda Demographic and Health Surveys. *Public Health Nutr* 2018;**21**:2915–28. doi:http://dx.doi.org/10.1017/S1368980018001982 |
| 8 | Ssewanyana S, Kasirye I. Causes of Health Inequalities in Uganda: Evidence from the Demographic and Health Surveys. *African Dev Rev* 2012;**24**:327–41. doi:10.1111/1467-8268.12007 |
| 9 | Shively GE. Infrastructure mitigates the sensitivity of child growth to local agriculture and rainfall in Nepal and Uganda. *Proc Natl Acad Sci U S A* 2017;**114**:903–8. doi:http://dx.doi.org/10.1073/pnas.1524482114 |
| 10 | Azzarri C, Zezza A, Haile B, *et al.* Does Livestock Ownership Affect Animal Source Foods Consumption and Child Nutritional Status? Evidence from Rural Uganda. *J Dev Stud* 2015;**51**:1034–59. doi:10.1080/00220388.2015.1018905 |
| 11 | Epstein A, Torres J, Glymour M, *et al.* Do Deviations from Historical Precipitation Trends Influence Child Nutrition? An Analysis from Uganda. *Am J Epidemiol* 2019;**188**:1953–60. doi:https://dx.doi.org/10.1093/aje/kwz179 |
| 12 | Assaf S, Gomez A, Juan C, *et al.* The association of deforestation and other environmental factors with child health and mortality. *DHS Anal Stud* Published Online First: 2018.http://invenio.unidep.org/invenio//record/21809/files/deforestation-and-child-mortality.pdf |
| 13 | Ickes SB, Hurst TE, Flax VL. Maternal literacy, facility birth, and education are positively associated with better infant and young child feeding practices and nutritional status among Ugandan children. *J Nutr* 2015;**145**:2578–86. doi:10.3945/jn.115.214346 |
| 14 | Engebretsen I, Tylleskar T, Wamani H, *et al.* Determinants of infant growth in Eastern Uganda: A community-based cross-sectional study. *BMC Public Health* 2008;**8**:1–12. doi:10.1186/1471-2458-8-418 |
| 15 | Kikafunda JK, Agaba E, Bambona A. Malnutrition amidst plenty: an assessment of factors responsible for persistent high levels of childhood stunting in food secure Western Uganda. *African J Food, Agric Nutr Dev* 2014;**14**:2088–113.http://www.ajfand.net/Volume14/No5/Agaba12570.pdf |
| 16 | Malembaka E, Tumwine J, Ndeezi G, *et al.* Effects of complementary feeding on attained height among lower primary school-aged children in Eastern Uganda: A nested prospective cohort study. *PLoS One* 2019;**14**:1–17. doi:10.1371/journal.pone.0211411 |
| 17 | Maxwell D, Levin C, Csete J. Does urban agriculture help prevent malnutrition? Evidence from Kampala. *Food Policy* 1998;**23**:411–24. doi:10.1016/S0306-9192(98)00047-5 |
| 18 | Wamani H, Tylleskar T, Astron A, *et al.* Mothers’ education but not fathers’ education, household assets or land ownership is the best predictor of child health inequalities in rural Uganda. *Int J Equity Health* 2004;**3**:1–8. doi:http://dx.doi.org/10.1186/1475-9276-3-1 |
| 19 | Muhoozi G, Atukunda P, Mwadime R, *et al.* Nutritional and developmental status among 6- to 8-month-old children in southwestern Uganda: A cross-sectional study. *Food Nutr Res* 2016;**60**:30270. doi:10.3402/fnr.v60.30270 |
| 20 | Amaral M, Herrin W, Gulere G. Using the Uganda National Panel Survey to analyze the effect of staple food consumption on undernourishment in Ugandan children. *BMC Public Health* 2018;**18**:1–12. doi:http://dx.doi.org/10.1186/s12889-017-4576-1 |
| 21 | Tiwari S, Skoufias E, Sherpa M. Shorter, Cheaper, Quicker, Better: Linking Measures of Household Food Security to Nutritional Outcomes in Bangladesh, Nepal, Pakistan, Uganda, and Tanzania. *World Bank Poverty Reducation Econonmic Manag Netw Poverty Reduct Equity Unit* 2013;:2–102. |
| 22 | Mackinnon J. Health as an Informational Good: The Determinants of Child Nutrition and Mortality During Political and Economic Recovery in Uganda. *Work Pap Ser Cent Study African Econ Univ Oxford* 1995;:1–30. |
| 23 | Kirk A, Kilic T, Carletto C. Composition of Household Income and Child Nutrition Outcomes Evidence from Uganda. *World Dev* 2018;**109**:452–69. doi:10.1016/j.worlddev.2017.03.023 |
| 24 | Vella V, Tomkins A, Borghesi A, *et al.* Determinants of stunting and recovery from stunting in Northwest uganda. *Int J Epidemiol* 1994;**23**:782–6. doi:10.1093/ije/23.4.782 |
| 25 | Mosites E, Rabinowitz P, Thumbi S, *et al.* The Relationship between Livestock Ownership and Child Stunting in Three Countries in Eastern Africa using National Survey Data. *PLoS One* 2015;**10**:1371–81. doi:http://dx.doi.org/10.1371/journal.pone.0136686 |
| 26 | Fierstein JL, Eliasziw M, Rogers BL, *et al.* Nonnative Cattle Ownership, Diet, and Child Height-for-Age: Evidence from the 2011 Uganda Demographic and Health Survey. *Am J Trop Med Hyg* 2017;**96**:74–82. doi:10.4269/ajtmh.16-0646 |
| 27 | Bridge A, Kipp W, Jhangri GS, *et al.* Nutritional status of young children in AIDS-affected households and controls in Uganda. *Am J Trop Med Hyg* 2006;**74**:926–31.http://ovidsp.ovid.com/ovidweb.cgi?T=JS&PAGE=reference&D=emed9&NEWS=N&AN=46774420 |
| 28 | Bukusuba J, Kaaya AN, Atukwase A. Predictors of Stunting in Children Aged 6 to 59 Months: A Case-Control Study in Southwest Uganda. *Food Nutr Bull* 2017;**38**:542–53. doi:http://dx.doi.org/10.1177/0379572117731666 |
| 29 | Hetherington JB, Wiethoelter AK, Negin J, *et al.* Livestock ownership, animal source foods and child nutritional outcomes in seven rural village clusters in Sub-Saharan Africa. *Agric Food Secur* 2017;**6**:3–11. doi:10.1186/s40066-016-0079-z |
| 30 | Habaasa G. An investigation on factors associated with malnutrition among underfive children in Nakaseke and Nakasongola districts, Uganda. *BMC Pediatr* 2015;**15**. doi:http://dx.doi.org/10.1186/s12887-015-0448-y |
| 31 | Vella V, Tomkins A, Borghesi A, *et al.* Determinants of child nutrition and mortality in north-west Uganda. *Bull World Health Organ* 1992;**70**:637–43.http://ovidsp.ovid.com/ovidweb.cgi?T=JS&PAGE=reference&D=emed4&NEWS=N&AN=22370551 |
| 32 | Ickes SB, Wu M, Mandel MP, *et al.* Associations between social support, psychological well-being, decision making, empowerment, infant and young child feeding, and nutritional status in Ugandan children ages 0 to 24 months. *Matern Child Nutr* 2018;**14**. doi:10.1111/mcn.12483 |
| 33 | Alderman H, Headey DD. The Nutritional Returns to Parental Education. *Int Food Policy Res Inst - Discuss Pap* 2014;**01379**.http://www.ifpri.org/sites/default/files/publications/ifpridp01379.pdf |
| 34 | Keats A. Women’s schooling, fertility, and child health outcomes: Evidence from Uganda’s free primary education program. *J Dev Econ* 2018;**135**:142–59. doi:10.1016/j.jdeveco.2018.07.002 |
| 35 | Wamani H, Astrom A, Peterson S, *et al.* Predictors of poor anthropometric status among children under 2 years of age in rural Uganda. *Public Health Nutr* 2006;**9**:320–6. doi:10.1079/PHN2006854 |
| 36 | Mukunya D, Kizito S, Orach T, *et al.* Knowledge of integrated management of childhood illnesses community and family practices (C-IMCI) and association with child undernutrition in Northern Uganda: A cross-sectional study. *BMC Public Health* 2014;**14**:976. doi:10.1186/1471-2458-14-976 |
| 37 | Turyashemererwa FM, Kikafunda JK, Agaba E. Prevalence of early childhood malnutrition and influencing factors in peri urban areas of Kabarole District, Western Uganda. *African J Food, Agric Nutr Dev* 2009;**9**:975–89.http://www.ajfand.net/Issue25/PDFs/Kinyata7030.pdf |
| 38 | Shively G, Hao J. A Review of Agriculture, Food Security and Human Nutrition Issues in Uganda. *Work Pap Dep Agric Econ Purdue Univ* 2012;**Working Pa**:1–42.http://ageconsearch.umn.edu/bitstream/135134/2/12-3Shively.Hao.pdf |
| 39 | Kabahenda MK, Andress EL, Nickols SY, *et al.* Promoting dietary diversity to improve child growth in less-resourced rural settings in Uganda. *J Hum Nutr Diet* 2014;**27**:143–51. doi:10.1111/jhn.12056 |
| 40 | Muhoozi G, Atukunda P, Diep L, *et al.* Nutrition, hygiene, and stimulation education to improve growth, cognitive, language, and motor development among infants in Uganda: A cluster-randomized trial. *Matern Child Nutr* 2018;**14**. doi:10.1111/mcn.12527 |
| 41 | Singla DR, Kumbakumba E, Aboud FE. Effects of a parenting intervention to address maternal psychological wellbeing and child development and growth in rural Uganda: A community-based, cluster-randomised trial. *Lancet Glob Heal* 2015;**3**:458–69. doi:10.1016/S2214-109X(15)00099-6 |
| 42 | Fadnes L, Nankabirwa V, Engebretsen I, *et al.* Effects of an exclusive breastfeeding intervention for six months on growth patterns of 4-5 year old children in Uganda: the cluster-randomised PROMISE EBF trial. *BMC Public Health* 2016;**16**:555. |
| 43 | Kajjura R, Veldman F, Kassier S. Effect of a novel supplementary porridge on the nutritional status of infants and young children diagnosed with moderate acute malnutrition in Uganda: a cluster randomised control trial. *J Hum Nutr Diet* 2019;**32**:295–302. doi:http://dx.doi.org/10.1111/jhn.12635 |
| 44 | Amegovu AK, Ochola S, Ogwok P, *et al.* Efficacy of sorghum peanut blend and corn soy blend plus in the treatment of moderate acute malnutrition in children aged 6-59 months in Karamoja, Uganda: a cluster randomized trial. *Nutr Diet Suppl* 2014;**6**:75–84. doi:10.2147/NDS.S64720 |
| 45 | FANTA-2. The Analysis of the Nutrition Situation in Uganda. Washington D.C.: 2010. |
| 46 | Arinaitwe E, Gasasira A, Verret W, *et al.* The association between malnutrition and the incidence of malaria among young HIV-infected and -uninfected Ugandan children: A prospective study. *Malar J* 2012;**11**. doi:http://dx.doi.org/10.1186/1475-2875-11-90 |
| 47 | Anand A, Nobhojit R. Transitioning toward Sustainable Development Goals: The Role of Household Environment in Influencing Child Health in Sub-Saharan Africa and South Asia Using Recent Demographic Health Surveys. *Front Public Heal* 2016;**4**. doi:10.3389/fpubh.2016.00087 |
| 48 | Lauer J, Duggan C, Ausman L, *et al.* Unsafe Drinking Water Is Associated with Environmental Enteric Dysfunction and Poor Growth Outcomes in Young Children in Rural Southwestern Ugand. *Am J Trop Med Hyg* 2018;**99**:1606–12. doi:http://dx.doi.org/10.4269/ajtmh.18-0143 |
| 49 | Olwedo M, Mworozi E, Bachou H, *et al.* Factors associated with malnutrition among children in internally displaced person’s camps, northern Uganda. *Afr Health Sci* 2008;**8**:244–52.http://ovidsp.ovid.com/ovidweb.cgi?T=JS&PAGE=reference&D=emed10&NEWS=N&AN=359389241 |
| 50 | Kikafunda J, Walker A, Collett D, *et al.* Risk factors for Early Childhood Malnutrition in Uganda. *Pediatrics* 1998;**102**:E45. doi:10.1542/peds.102.4.e4 |
| 51 | Jones A, Ickes S, Smith L, *et al.* World Health Organization infant and young child feeding indicators and their associations with child anthropometry: a synthesis of recent findings. *Matern Child Nutr* 2014;**10**:1–17. doi:10.1111/mcn.12070 |
| 52 | Nahlomo A, Iverson P, Rukundo PM, *et al.* Realization of the right to adequate food and the nutritional status of land evictees: A case for mothers/caregivers and their children in rural Central Uganda. *BMC Int Health Hum Rights* 2018;**18**:1–13. doi:10.1186/s12914-018-0162-6 |
| 53 | Yeudall F, Sebastian R, Cole D, *et al.* Food and nutritional security of children of urban farmers in Kampala, Uganda. *Food Nutr Bull* 2007;**28**:S237–46.https://www.scopus.com/inward/record.uri?eid=2-s2.0-34250322537&partnerID=40&md5=bb0faf9694c2c817b6c31c00c1f1cf52 |
| 54 | Mor S, Tumwine J, Naumova E, *et al.* Microsporidiosis and Malnutrition in Children with Persistent Diarrhea, Uganda. *Emerg Infect Dis* 2009;**15**:49–52. doi:http://dx.doi.org/10.3201/eid1501.071536 |
| 55 | Cabanero-Verzosa C. Counting on Commmunication: the Uganda Nutrition and Early Childhood Development Project. *World Bank Work Pap* 2005;**59**. doi:10.1596/978-0-8213-6268-6 |
| 56 | UNICEF, Cardiff University, UBOS, *et al.* Views of the Public. 2019. https://www.unicef.org/uganda/media/5136/file/Multidimensional child poverty and deprivation in Uganda Report Volume 2.pdf |

## **Supplementary Figure 3A:** Victora curves for children under-5 from 2000 to 2016 with linear splines.


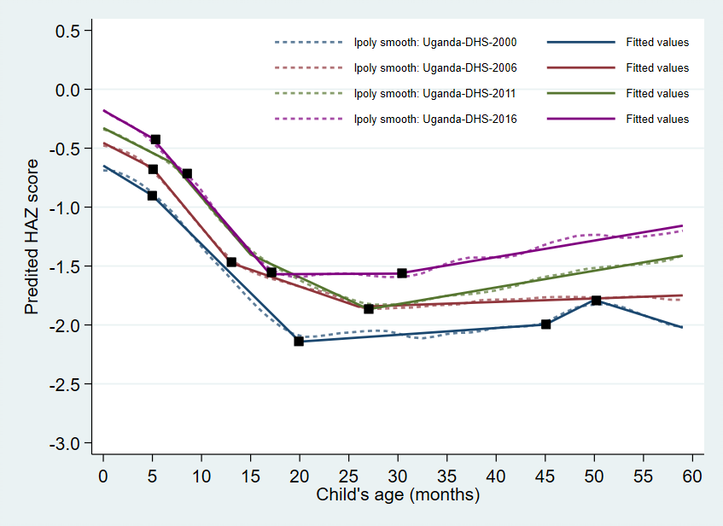


**Supplementary Figure 3B:** Spline analysis of inflection points of change in the slope of HAZ, 2000.


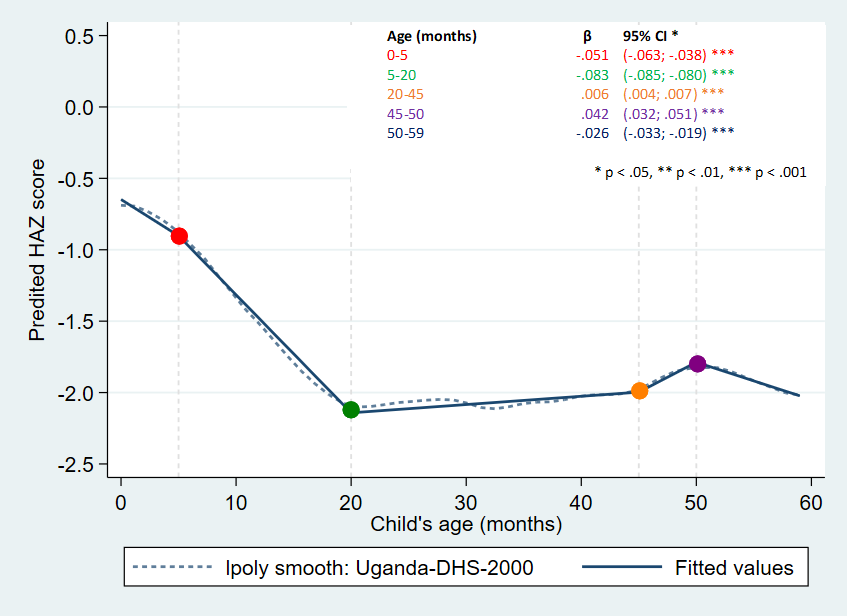


**Supplementary Figure 3C:** Spline analysis of inflection points of change in the slope of HAZ, 2006.


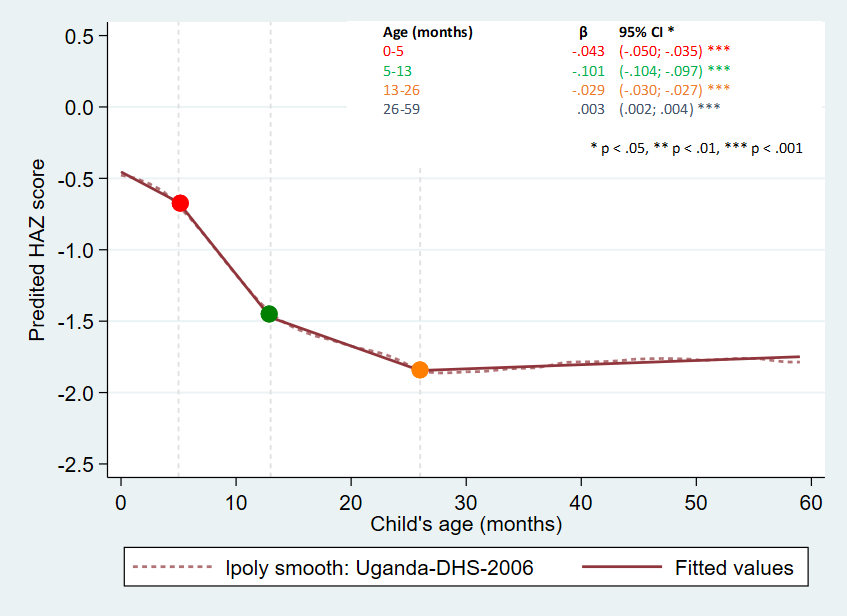


**Supplementary Figure 3D:** Spline analysis of inflection points of change in the slope of HAZ, 2011.


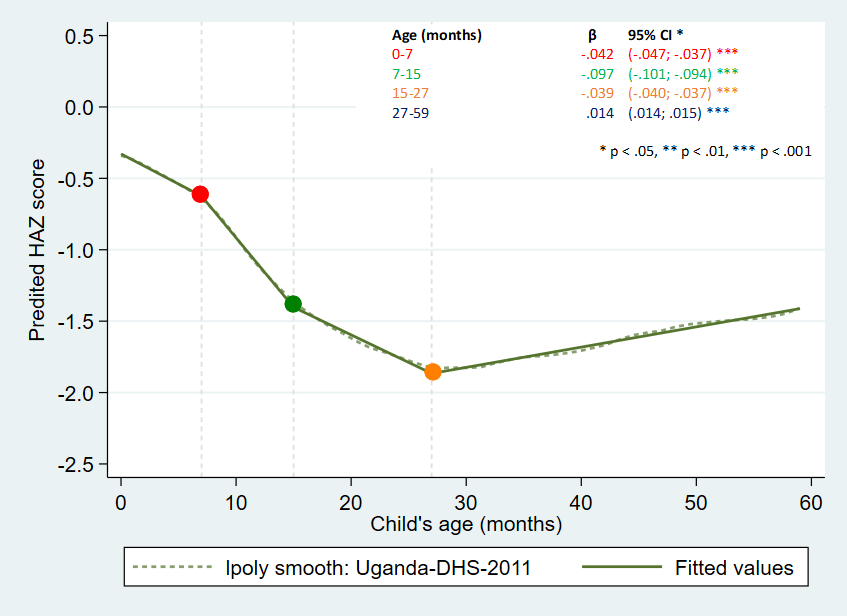


**Supplementary Figure 3E:** Spline analysis of inflection points of change in the slope of HAZ, 2016.


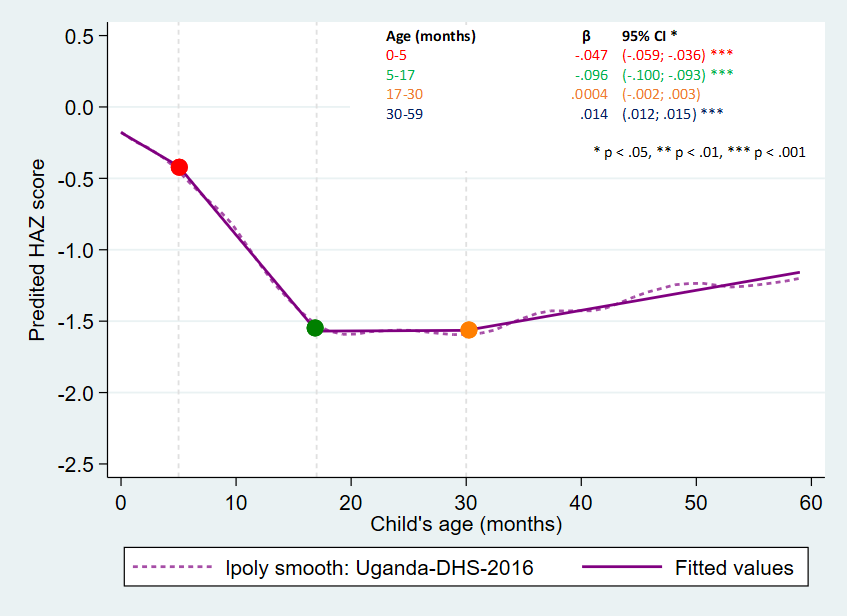


## **Supplementary Figure 4A:** Regional maps of stunting decline between 2000 and 2016.

2000


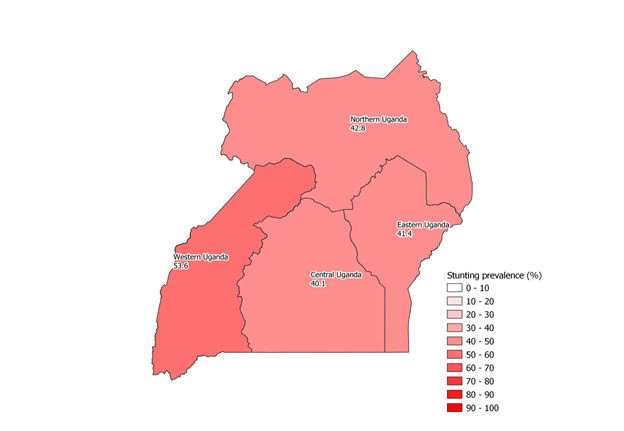


2006


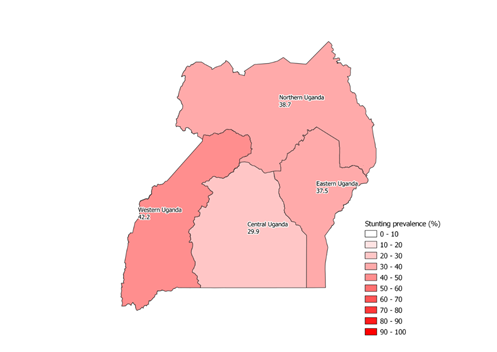


2011


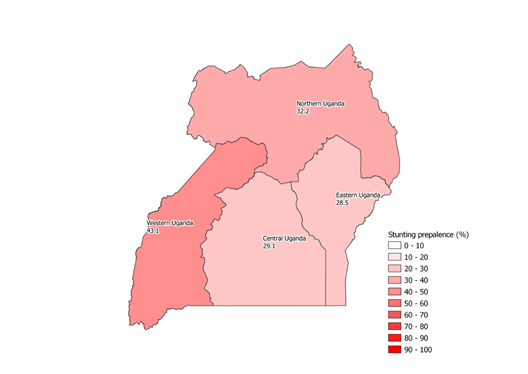


2016


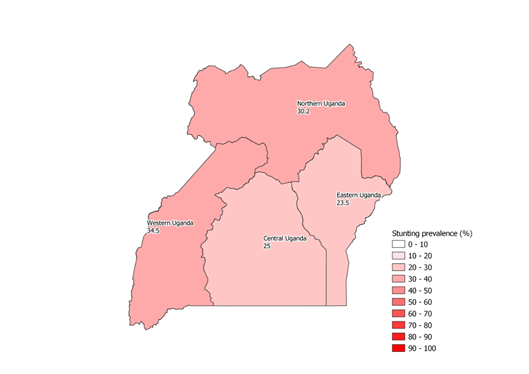


## **Supplementary Figure 4B:** Average annual rate of change and compound annual growth rate (decline) of stunting among children under-5, by region.


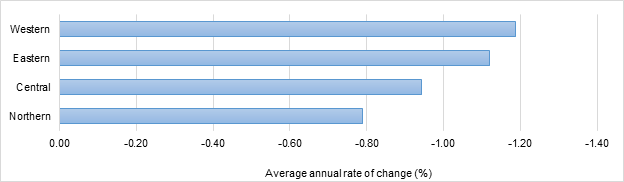


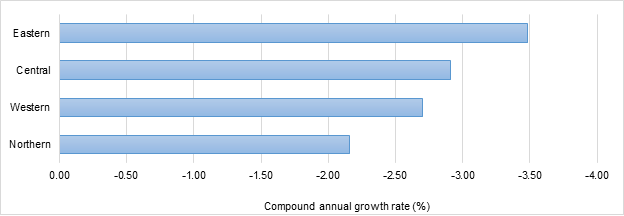


**Supplementary Figure 5:** District-level stunting prevalence in 2016.


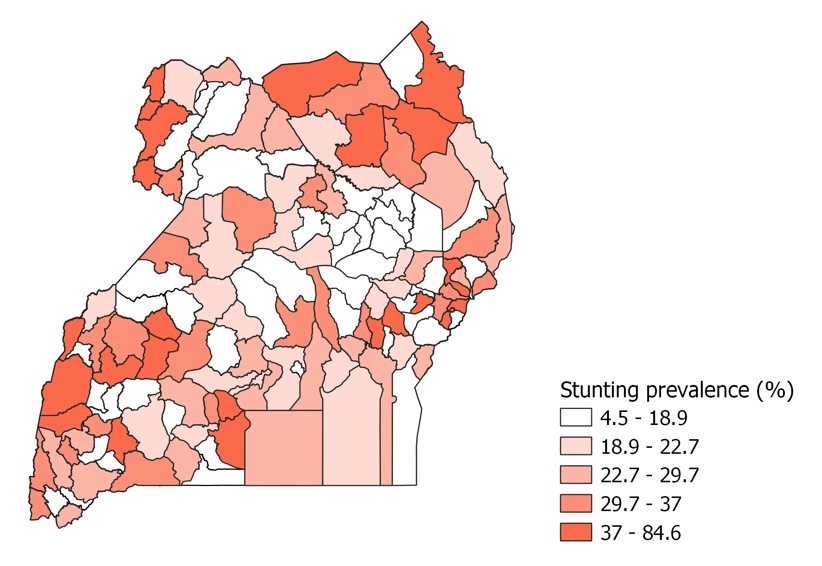


**Supplementary Figure 6A:** Change in absolute slope index of inequality (SII) by year in Uganda.


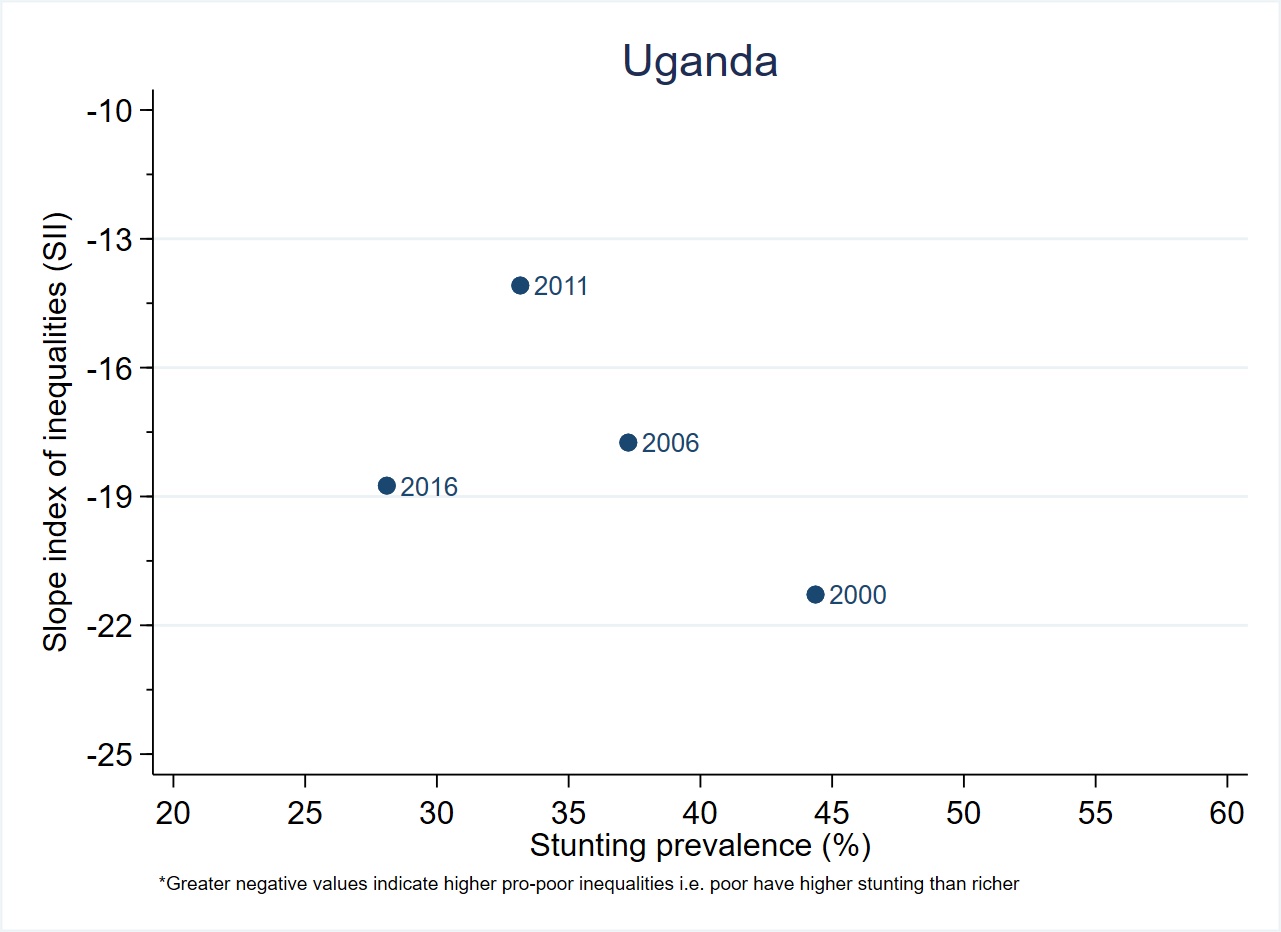


**Supplementary Figure 6B:** Change in relative concentration index (CIX) by year in Uganda.


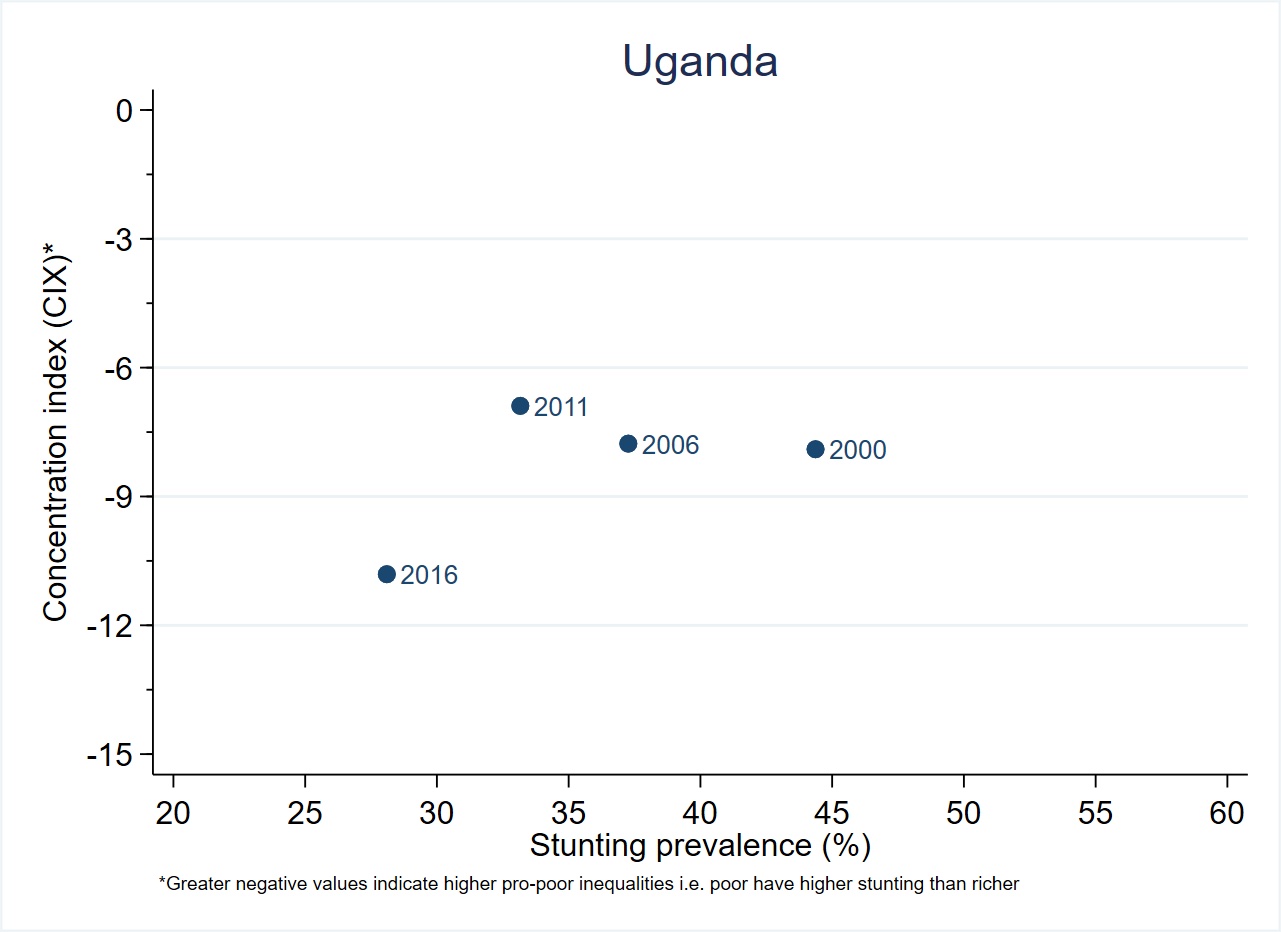


## **Supplementary Figure 7:** Change in district-level malaria prevalence (2-10 years) from 2000 to 2016 and district-level stunting in 2016.


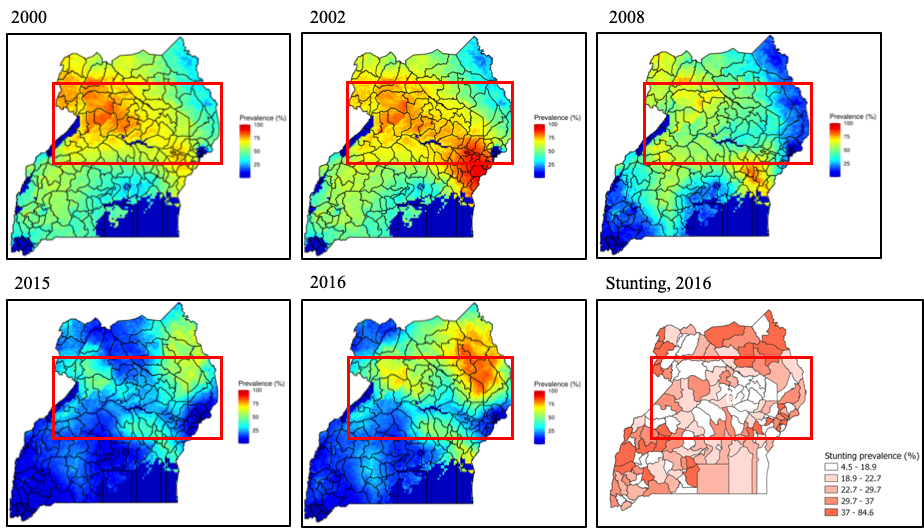


## **Supplementary Figure 8A:** District-level stunting prevalence versus household ownership of insecticide-treated bed-nets, 2016. Correlation coefficient (R)= - 0.165**.**


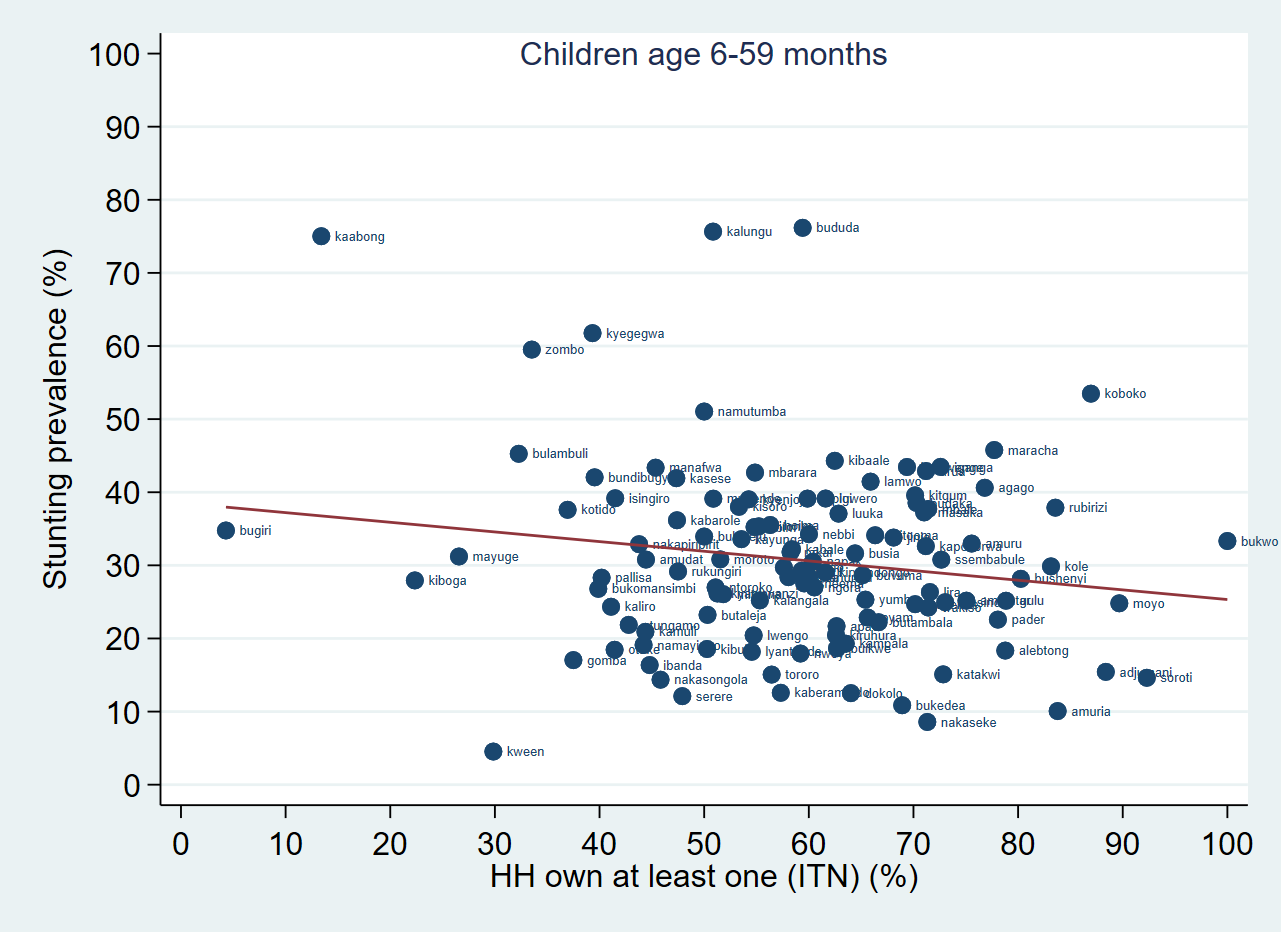


## **Supplementary Figure 8B**: District-level stunting prevalence versus children sleeping under a bed-net, 2016. Correlation coefficient (R)= -0.139.


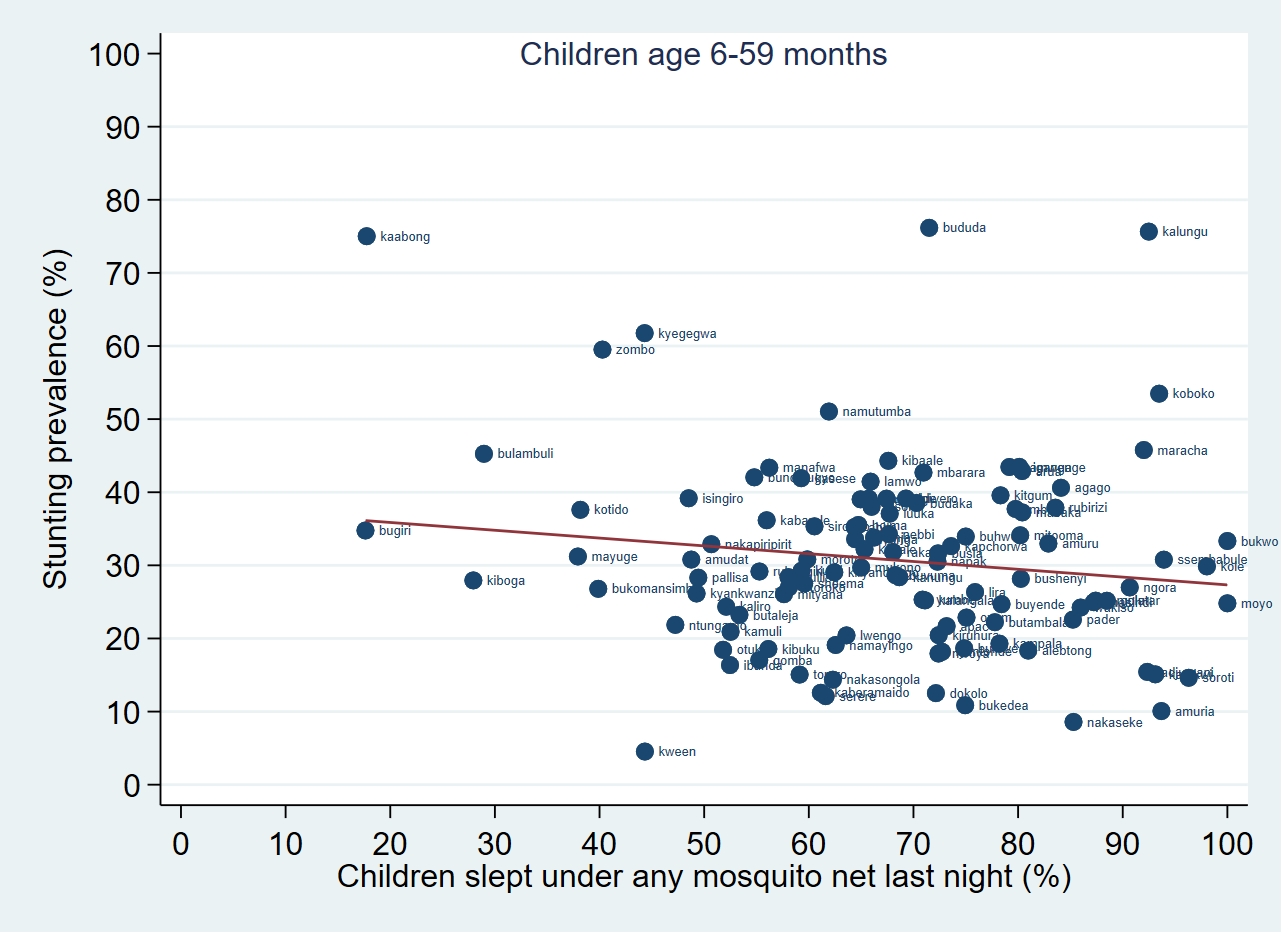


## **Supplementary Table 2**: Sample size by survey based on index child with available anthropometry data.

| **Year of Demographic and Health Survey** | | | | |
| --- | --- | --- | --- | --- |
| **Age group** | **2000** | **2006** | **2011** | **2016** |
| <5 y | 3234 | 1447 | 1302 | 2968 |
| <36 mo | 2746 | 1208 | 1081 | 2327 |
| 24+ mo | 1051 | 493 | 453 | 1226 |
| 6–23 mo | 1691 | 726 | 635 | 1291 |
| <6 mo | 492 | 228 | 214 | 451 |

## **Supplementary Table 3:** Decomposition analysis for children under-5 from 2000-2016.

| **Factors** | **Estimated coefficient** | **Mean difference (2016 - 2000)** | **Predicted change in HAZ** | | **Share of predicted change (%)** |
| --- | --- | --- | --- | --- | --- |
| HAZ Score | - | 0.55 | 0.45 | 81.6% | |
| Wealth index | 0.037 | 0.57 | 0.021 | 4.8% | |
| Mother education | 0.035 | 2.24 | 0.077 | 17.3% | |
| Father education | 0.018 | 1.42 | 0.026 | 5.8% | |
| Women empowerment of health care | 0.114 | 0.09 | 0.011 | 2.4% | |
| Skilled birth attendant | 0.069 | 0.55 | 0.038 | 8.4% | |
| Antenatal care visits 4+ | 0.133 | 0.18 | 0.024 | 5.3% | |
| Open defecation | -0.123 | -0.10 | 0.012 | 2.7% | |
| Water source pipe | 0.186 | 0.11 | 0.020 | 4.5% | |
| Household crowding | 0.034 | -0.32 | -0.011 | -2.4% | |
| Households had mosquito bed net for sleeping | 0.271 | 0.71 | 0.194 | 43.4% | |
| Diarrhea | -0.167 | -0.01 | 0.001 | 0.3% | |
| Early age pregnancy | -0.180 | -0.01 | 0.003 | 0.6% | |
| Maternal anemia | -0.134 | -0.07 | 0.009 | 2.1% | |
| Maternal BMI | 0.044 | 1.31 | 0.058 | 13.0% | |
| Maternal height | 0.055 | 0.60 | 0.033 | 7.5% | |
| Inter-pregnancy interval (in months) | 0.003 | 4.14 | 0.011 | 2.4% | |
| Others* | - | - | -0.081 | -18.1% | |

*Others includes region, child age, and child sex.

## **Supplementary Table 4:** Decomposition analysis for children <6 months from 2000-2016.

| **Factors** | **Estimated coefficient** | **Mean difference (2016 - 2000)** | **Predicted change in HAZ** | **Share of predicted change (%)** |
| --- | --- | --- | --- | --- |
| HAZ Score | - | 0.50 | 0.70 | 138.7% |
| Mother year of education | 0.040 | 2.25 | 0.09 | 13.0% |
| Women empowerment for health care | 0.233 | 0.14 | 0.03 | 4.6% |
| Skilled birth attendant | 0.087 | 0.56 | 0.05 | 7.0% |
| Antenatal care visits 4+ | 0.287 | 0.19 | 0.05 | 7.7% |
| Piped water | 0.421 | 0.09 | 0.04 | 5.3% |
| Household with mosquito bed net | 0.433 | 0.72 | 0.31 | 44.4% |
| ARI | -0.304 | -0.13 | 0.04 | 5.6% |
| Maternal anemia | -0.315 | 0.02 | -0.01 | -0.7% |
| Maternal BMI | 0.058 | 1.17 | 0.07 | 9.7% |
| Maternal height | 0.044 | 0.64 | 0.03 | 4.0% |
| Inter-pregnancy interval (in months) | -0.009 | 4.30 | -0.04 | -5.6% |
| Others* | - | - | 0.03 | 5.0% |

*Others includes region, child age, and child sex.

## **Supplementary Table 5**: Decomposition analysis for children 6-23 months from 2000-2016.

| **Factors** | **Estimated coefficient** | **Mean difference (2016 - 2000)** | **Predicted change in HAZ** | **Share of predicted change (%)** |
| --- | --- | --- | --- | --- |
| HAZ Score | - | 0.53 | 0.59 | 111.9% |
| Wealth index | 0.035 | 0.57 | 0.02 | 3.3% |
| Mother education | 0.023 | 2.50 | 0.06 | 9.7% |
| Father education | 0.023 | 1.46 | 0.03 | 5.7% |
| Women empowerment for health care | 0.171 | 0.23 | 0.04 | 6.6% |
| Complementary feeding | -0.174 | -0.04 | 0.01 | 1.1% |
| DPT vaccination | 0.188 | 0.20 | 0.04 | 6.4% |
| Skilled birth attendant | 0.082 | 0.57 | 0.05 | 7.9% |
| Antenatal care visits 4+ | 0.117 | 0.21 | 0.02 | 4.2% |
| Reduced open defecation | -0.227 | -0.09 | 0.02 | 3.6% |
| Household crowding | 0.029 | -0.12 | 0.00 | -0.6% |
| Child slept under mosquito bet net last night | 0.317 | 0.64 | 0.20 | 34.0% |
| Diarrhea | -0.115 | 0.03 | 0.00 | -0.6% |
| Maternal anemia | -0.100 | -0.08 | 0.01 | 1.4% |
| Maternal BMI | 0.035 | 1.12 | 0.04 | 6.6% |
| Maternal height | 0.054 | 0.75 | 0.04 | 6.9% |
| Inter-pregnancy interval (in months) | 0.003 | 5.18 | 0.02 | 2.5% |
| Others* | - | - | 0.01 | 1.4% |

*Others includes region, child age, and child sex.

## **Supplementary Table 6:** Decomposition analysis for children 24+ months from 2000-2016.

| **Factors** | **Estimated coefficient** | **Mean difference (2016 - 2000)** | **Predicted change in HAZ** | **Share of predicted change (%)** |
| --- | --- | --- | --- | --- |
| HAZ Score | - | 0.684 | 0.45 | 65.9% |
| Wealth index | 0.055 | 0.572 | 0.03 | 7.0% |
| Mother education | 0.045 | 1.895 | 0.09 | 18.9% |
| Skilled birth attendant | 0.047 | 0.517 | 0.02 | 5.4% |
| Antenatal care visits 4+ | 0.135 | 0.125 | 0.02 | 3.7% |
| Household with mosquito net | 0.266 | 0.710 | 0.19 | 41.9% |
| Maternal age | 0.021 | 0.269 | 0.01 | 1.2% |
| Maternal BMI | 0.044 | 1.421 | 0.06 | 13.7% |
| Maternal height | 0.059 | 0.346 | 0.02 | 4.5% |
| Inter-pregnancy interval (in months) | 0.005 | 2.936 | 0.01 | 3.3% |
| Others* | - | - | 0.00 | 0.3% |

*Others includes region, child age, and child sex.

## **Supplementary Table 7:** Difference-in-difference multivariable regression analysis for children under-5 from 2000-2016.

| Domain/Indicator | Outcome = HAZ | | | |
| --- | --- | --- | --- | --- |
|  | (Height for age z-score for under-5 children) | | | |
|  | Period 2000 to 2016 in Uganda | | | |
|  | Bivariate regression coefficient | | Final multivariable regression coefficient* | |
|  |  | β estimate (95% CI) |  | β estimate (95% CI) |
|  |  | *p*-value |  | *p*-value |
|  |  |  |  |  |
| **Distal level** | | | | |
| **Basic causes & Income poverty** | | | | |
| Wealth Index (nine components using PCA) |  | 0.044 (0.033, 0.055) |  | 3.16 (-0.441, 6.762) |
| (0 - 10) |  | <0.001 |  | 0.085 |
|  |  |  |  |  |
| Wealth Index#Year |  | -0.002 (-0.0036, -0.0004) |  | -0.0016 (-0.0034, 0.0002) |
| (0 - 10) |  | 0.013 |  | 0.089 |
|  |  |  |  |  |
| Mother years of schooling |  | 0.055 (0.045, 0.064) |  | 0.034 (0.021, 0.046) |
|  |  | <0.001 |  | <0.001 |
|  |  |  |  |  |
| Maternal education # year |  | 0 (-0.002, 0.001) |  |  |
|  |  | 0.478 |  |  |
|  |  |  |  |  |
| Father years of schooling |  | 0.046 (0.037, 0.055) |  | 2.942 (0.253, 5.631) |
|  |  | <0.001 |  | 0.032 |
|  |  |  |  |  |
| Father education # Year |  | -0.0014 (-0.0027, -0.0001) |  | -0.0015 (-0.0028, -0.0001) |
|  |  | 0.041 |  | 0.033 |
|  |  |  |  |  |
| HH purchase |  | 0.02 (-0.062, 0.102) |  | 0.126 (0.049, 0.203) |
| (% women decide on large HH purchase) |  | 0.627 |  | 0.001 |
|  |  |  |  |  |
| HH purchase # year |  | 0.014 (0.0022, 0.0258) |  |  |
|  |  | 0.02 |  | -- |
|  |  |  |  |  |
| HC purchase |  | 0.041 (-0.04, 0.121) |  |  |
| (% women who decide on healthcare purchase) |  | 0.322 |  | -- |
|  |  |  |  |  |
| HC purchase # year |  | -0.0003 (-0.0124, 0.0118) |  |  |
|  |  | 0.956 |  | -- |
|  |  |  |  |  |
| **Intermediate level** | | | | |
| **Inadequate care and health services** | | | | |
| Skilled birth attendant |  | 0.275 (0.189, 0.362) |  | 0.082 (-0.013, 0.178) |
| (% women with SBA) |  | <0.001 |  | 0.09 |
|  |  |  |  |  |
| Skilled birth attendant # year |  | -0.006 (-0.019, 0.007) |  |  |
|  |  | 0.341 |  | -- |
|  |  |  |  |  |
| Antenatal care |  | 0.172 (0.097, 0.246) |  | 0.116 (0.039, 0.194) |
| (% women with at least 4 visits) |  | <0.001 |  | 0.003 |
|  |  |  |  |  |
| Antenatal care # Year |  | -0.008 (-0.019, 0.003) |  |  |
|  |  | 0.136 |  | -- |
|  |  |  |  |  |
| Place of delivery |  | 0.276 (0.197, 0.355) |  |  |
| (% of delivery at medical facility) |  | <0.001 |  | -- |
|  |  |  |  |  |
| Place of delivery # year |  | -0.007 (-0.0186, 0.0046) |  |  |
|  |  | 0.238 |  | -- |
|  |  |  |  |  |
| **Unhealthy household environment** | | | | |
| Urbanization |  | 0.39 (0.285, 0.496) |  | 0.224 (0.102, 0.347) |
| (% of urban population) |  | <0.001 |  | <0.001 |
|  |  |  |  |  |
| Urbanization # Year |  | -0.0226 (-0.0364, -0.0087) |  |  |
|  |  | 0.001 |  | -- |
|  |  |  |  |  |
| Open defecation |  | -0.168 (-0.28, -0.056) |  | -0.129 (-0.248, -0.009) |
| (% population) |  | 0.003 |  | 0.035 |
|  |  |  |  |  |
| Open defecation # Year |  | 0.008 (-0.0079, 0.0239) |  |  |
|  |  | 0.324 |  | -- |
|  |  |  |  |  |
| Water source - piped |  | 0.267 (0.165, 0.37) |  |  |
| (% of population) |  | <0.001 |  | -- |
|  |  |  |  |  |
| Water source - piped # Year |  | -0.0156 (-0.0305, -0.0006) |  |  |
|  |  | 0.041 |  | -- |
|  |  |  |  |  |
| Household crowding |  | 0.0215 (0.008, 0.0349) |  | 0.032 (0.016, 0.047) |
| (Number of household members) |  | 0.002 |  | <0.001 |
|  |  |  |  |  |
| Household crowding # Year |  | -0.00003 (-0.00191, 0.00186) |  |  |
|  |  | 0.978 |  | -- |
|  |  |  |  |  |
| Mass media |  | 0.591 (0.388, 0.7941) |  |  |
| (% of women exposure to mass media at least once a week) |  | <0.001 |  | -- |
|  |  |  |  |  |
| Mass media # Year |  | -0.02008 (-0.04813, 0.00798) |  |  |
|  |  | 0.161 |  | -- |
|  |  |  |  |  |
| HH with mosquito net |  | 0.332 (0.243, 0.421) |  | 29.042 (-0.991, 59.075) |
| (% of Households that own at least one mosquito net) |  | <0.001 |  | 0.058 |
|  |  |  |  |  |
| HH with mosquito net # Year |  | -0.022 (-0.0351, -0.0088) |  | -0.014 (-0.029, 0.001) |
|  |  | 0.001 |  | 0.06 |
|  |  |  |  |  |
| Child slept under mosquito net |  | 0.32 (0.229, 0.411) |  |  |
| (% of Children under-5 who slept under any mosquito net last night) |  | <0.001 |  | -- |
|  |  |  |  |  |
| Child slept under mosquito net # Year |  | -0.0235 (-0.0376, -0.0094) |  |  |
|  |  | 0.001 |  | -- |
|  |  |  |  |  |
| Women received IPTP |  | 0.061 (-0.041, 0.163) |  |  |
|  |  | 0.24 |  | -- |
|  |  |  |  |  |
| Women received IPTP # Year |  | -0.0129 (-0.0302, 0.0043) |  |  |
|  |  | 0.141 |  | -- |
|  |  |  |  |  |
| **Proximal level** | | | | |
| **Disease** | | | | |
| Acute Respiratory incidence/ reports |  | -0.108 (-0.206, -0.01) |  | -28.291 (-57.71, 1.127) |
| (% under-5 population within last 2 weeks) |  | 0.032 |  | 0.059 |
|  |  |  |  |  |
| Acute Respiratory incidence # Year |  | 0.019 (0.004, 0.034) |  | 0.014 (-0.001, 0.029) |
|  |  | 0.011 |  | 0.06 |
|  |  |  |  |  |
| Diarrhea incidence/reports |  | -0.1216 (-0.2002, -0.0429) |  | -0.212 (-0.292, -0.131) |
| (% under-5 population within last 2 weeks) |  | 0.002 |  | <0.001 |
|  |  |  |  |  |
| Diarrhea incidence # Year |  | 0.01 (-0.001, 0.021) |  |  |
|  |  | 0.077 |  | -- |
|  |  |  |  |  |
| **Maternal characteristics** | | | | |
| Age |  | -0.0029 (-0.0082, 0.0025) |  |  |
| (Mean, mothers 15-49) |  | 0.296 |  | -- |
|  |  |  |  |  |
| Age# Year |  | 0.0003 (-0.0005, 0.001) |  |  |
|  |  | 0.487 |  |  |
|  |  |  |  |  |
| Index births within last 5 years |  | -0.29 (-0.41, -0.171) |  | -0.24 (-0.389, -0.09) |
| (% mothers <18 years) |  | <0.001 |  | 0.002 |
|  |  |  |  |  |
| Index birth within last 5 years # Year |  | -0.002 (-0.018, 0.015) |  |  |
|  |  | 0.854 |  | -- |
|  |  |  |  |  |
| Index births within last 5 years |  | -0.034 (-0.135, 0.067) |  |  |
| (% mothers >= 35 years) |  | 0.515 |  | -- |
|  |  |  |  |  |
| Index birth within last 5 years # Year |  | 0.004 (-0.01, 0.018) |  |  |
|  |  | 0.593 |  | -- |
|  |  |  |  |  |
| Anemia (WRA) |  | -0.136 (-0.208, -0.064) |  | -0.1 (-0.174, -0.027) |
| (% of women with anemia) |  | <0.001 |  | 0.007 |
|  |  |  |  |  |
| Maternal anemia # Year |  | 0.0088 (-0.0013, 0.019) |  |  |
|  |  | 0.088 |  | -- |
|  |  |  |  |  |
| BMI level |  | 0.04 (0.03, 0.05) |  | 0.044 (0.033, 0.056) |
| (Mean mothers 15-49 years) |  | <0.001 |  | <0.001 |
|  |  |  |  |  |
| BMI level # Year |  | -0.00035 (-0.00173, 0.00103) |  |  |
|  |  | 0.615 |  | -- |
|  |  |  |  |  |
| Height |  | 0.054 (0.048, 0.059) |  | 0.056 (0.049, 0.063) |
| (Mean mothers 15-49 years) |  | <0.001 |  | <0.001 |
|  |  |  |  |  |
| Height # Year |  | 0.0004 (-0.0004, 0.0013) |  |  |
|  |  | 0.327 |  | -- |
|  |  |  |  |  |
| Parity |  | -0.012 (-0.027, 0.002) |  |  |
| (Total fertility rate) |  | 0.089 |  | -- |
|  |  |  |  |  |
| Parity # Year |  | 0.0005 (-0.0016, 0.0025) |  |  |
|  |  | 0.667 |  | -- |
|  |  |  |  |  |
| Inter-pregnancy interval |  | 0.003 (0.001, 0.004) |  | 0.002 (0.001, 0.004) |
| (in months) |  | 0.002 |  | 0.008 |
|  |  |  |  |  |
| Inter-pregnancy intervals # Year |  | 0.0002 (0, 0.0004) |  |  |
|  |  | 0.098 |  | -- |
|  |  |  |  |  |
| **Time** | | | | |
| **Time** | | | | |
| Year |  | 0.034 (0.028, 0.04) |  | 0.028 (0.013, 0.043) |
|  |  | <0.001 |  | <0.001 |
|  |  |  |  |  |

Red text indicates significant time*covariate interaction terms.

Notes: Variables significant at p<0.20 in bivariate analysis were entered into linear models. ^a^Level 3multivariable model includes all statistically significant (p<0.10) distal variables as listed. ^b^Level 2 multivariable model includes level 3 model all statistically significant (p<0.10) intermediate variables as listed. ^c^Level 1 multivariable model includes level 3 model + 2 model + all statistically significant (p<0.10) proximal variables as listed.

## **Supplementary Table 8:** Themes and supporting quotes from key informant interviews and focus group discussions.

| **THEME** | **QUOTE** |
| --- | --- |
| **Political Commitment and Multi-Sectoral Action** | “*The political will has really been very supportive of nutrition to the extent that if you look at the Uganda Nutrition Action Plan which aimed at encouraging all the key sectors to mainstream Nutrition interventions in their plans was signed by the president of Uganda”.* [KII, MoH]  *“Financial investment will affect human resources, affect commodities and we see this very clearly in the budget of the Ministry of Health. If you tried to track nutrition budget in the Ministry of Health you will be very disappointed because you can’t see it anywhere! The policies are there but require investment to be translated into action but now with the multisectoral approaches all departments, ministries and agencies are required to indicate a specific budget for nutrition.”* [KII, National level] |
| **Conflict** | “*Before the 1990s, the political stability was worse… apparently, conflicts have reduced, we have improvement in the health indicators. Food insecurity has declined; there are more public health interventions because of the political stability*.” [KII, MAAIF]  *“Previously in 1996 and 1997, there was political instability that limited us to get food, since that time, we don’t harvest well, and we were suffering because you could not give your children enough food… we had some limitations towards getting food.”* [FGD, mother of child born 1995-2000, Kasese] |
| **Climate Change** | *“These days, there is change in climate, you find there are many floods that take away crops and you even harvest nothing, sometimes even rain can disappear and you face drought which destroys all our crops. So these days we no longer store foods like the in past years which has affected our children’s nutrition.”* [FGD, mother of child born 2011-2016, Kasese] |
| **Poverty** | *“If you travel across Uganda then you see the inequality; in some areas we are doing well while in others, we are not doing well at all; poverty is still the biggest problem.”* [KII, Academia] |
| **Education** | “*Now we have the Universal primary and secondary education and the people are informed about what to feed on during the radio talk shows and from the extension workers.*” [KII, Serere]  “*The highly educated people have no time for their children because they have a constraint called time, they are so busy and children age less than two years they need to be breastfed. However, these days women don’t breastfeed and yet breast feeding is very key, they start giving formula feeds at an early age, so issues of hygiene greatly affect their children and they routinely get diarrheal infection.*” [KII, Kasese] |
| **Women’s Empowerment** | “*There has been improvement in child nutrition from 2010 up to date. The nutrition status in the past years [in 90’s] was not good because all the powers were in the hands of men but now when you look back 20 years ago and compare now, women are economically empowered and they can provide their children with nutritious food than before. So it is like now 10 years when we have been economically empowered*.” [FGD, mother of child born 2011-2016, Serere] |
| **Malaria Reduction** | *The ministry of health has addressed the issue of mosquito net coverage among under five children, which has reduce on malaria incidence in the region because sick children do not grow well. In addition, we have the community distribution of drugs so children to provide community treatment within 24 hours. The reduction in malaria occurrence coupled with appropriate feeding practices has had a significant improvement on the development of the children.”* [KII, Serere]  “*Malaria is still our biggest problem. There was a very effective/ vibrant malaria control program between the 2001 and 2016 surveys, which could have contributed to a sharp decline in anemia in both women and children under five; but in 2016 we saw an upward trend. So the preventive services being done on the control of malaria should continue.”*  [KII, MoH] |
| **Water, Sanitation, and Hygiene** | *“There is a problem of land whereby like in our centre where I stay, you find we use only one latrine because of inadequate space, the latrine is always dirty because it is used by many people including children and adults, … even if you have your own latrine, still people break the padlock and use it and they just defecate anywhere around your latrine, you find the hygiene and sanitation is poor. Even when a child stays in a dirty environment, the child cannot grow well.”* [FGD, mother of child born 1995-2000, Kasese] |
| **Agricultural Practices** | *“There is a positive change because one can now grow food crops for home consumption and at the same time grow commercial foods such as the vitamin A rich sweet potatoes, and when you get money from selling agricultural products, you can buy goats which you can rare from your home and later sell to earn you income which supplement on the children’s diet.”* [FGD, mother of child born 2011-2016, Serere]  *“Food has reduced in both quantity and quality because of the climate change, the soils are becoming exhausted, and people are very poor at using fertilizers, so it becomes worse in the mountainous places where soil is carried away by soil erosion or by water, the top soil which is fertile is washed away every now and then.”* [KII, Kasese] |
| **Adolescent Pregnancies** | *“The young girls who are giving birth to many children; you find that a child is not even 2 months and the mother is again pregnant, in this case, it will be very difficult for such a woman to raise children well.”* [FGD, mother of child born 2011-2016, Serere]  *“The situation among adolescents regarding early marriages including dropping out of school for marriage at an early has worsened in the Eastern region which has a big influence on stunting reduction. Moreover, there are very many young girls who are not capable of carrying a womb as early as 15 years and sustaining that pregnancy for 9 months. Thus, the child is born when it is too small and the mother is still naïve in bringing up the child which may result its growth retardation.”* [KII, Makerere University] |
| **Breastfeeding** | *“Exclusive breastfeeding has reduced especially in the urban areas because of urbanization than it used to be in the past. Nonetheless, in rural areas mothers endeavor to breastfeed exclusively because they are able to carry along their children to the place of work such as garden unlike their counterpart in urban setting.”* [KII, National level]  *“The breastfeeding mothers also know, they know what is supposed to be done but knowing and practicing is another thing, there are a number of issues around breastfeeding, most of them complain of lack of enough milk, because they don’t eat enough. Secondly, they are very busy so they don’t get enough time to breastfeed their children. For instance, they are going for markets, thinking of money, so exclusive breastfeeding is really very low in the area where I work in Kisinga.”* [KII, Kasese] |
| **Burden of Child Diseases** | *“We are seeing some of these other conditions but HIV still causes malnutrition but not as much as it used to. In addition, where there is high reduction in diarrhea you see some figures of stunting going down. One of the diseases that have a major impact in nutrition is malaria because of anemia. However, we no longer have many people getting malaria.”* [KII, WHO] |
| **Dietary Intake** | *“We have the knowledge to feed our children well because we have been educated by our nurses and other organizations to feed our children well but now, you plant crops and floods take away the crops. You find you don’t have beans, groundnuts and other foods, you find yourself eating the same food.”* [FGD, mother of child born 2011-2016, Kasese |

FGD: focus group discussion; KII: key informant interview; MAAIF: Ministry of Agriculture, Animal Industries, and Fisheries; MoH: Ministry of Health; WHO: World Health Organization.

## **Supplementary Table 9:** Policy and program review findings.

| *NATIONAL-LEVEL POLICIES/PROGRAMS* | | |
| --- | --- | --- |
| Universal Salt Iodization Policy (1993) | Description | The Universal Salt Iodization (USI) Policy was enacted in 1993 that required all salt for human and livestock consumption in Uganda to be adequately iodized with potassium iodate. |
|  | Classification | Indirect Health Sector Intervention |
| Uganda Water Action Plan (1995) | Description | The Uganda Water Action Plan was prepared to facilitate an operational and sustainable water resources management. The plan identified key water resources management issues, and oversaw the development of the National Water Policy and legislative framework for the protection and development of Uganda’s water resources. |
|  | Classification | Indirect Other Sectoral Strategy |
| Water Statute (1995) | Description | The Water Statute was established in 1995 with the aim of ensuring a clean, safe, and sufficient water supply for domestic use by the Ugandan population. The statute was reviewed in 1997 under The Water Act. |
|  | Classification | Indirect Other Sectoral Strategy |
| Baby Friendly Hospital Initiative (1996) | Description | The Baby Friendly Hospital Initiative (BFHI) was introduced to improve hospital routines and procedures to become more supportive of the successful initiation and continuation of optimal breastfeeding practices. Uganda implemented an additional six steps, including mandatory vitamin A supplementation to mothers right after delivery and mandatary BCG and polio vaccines for newborns before discharge. |
|  | Classification | Direct Health Sector Nutrition Intervention |
| The Food and Drugs (Marketing of Infant and Young Child Feeding) Regulations (1997) | Description | These guidelines were enacted in 1997 to regulate the marketing of infant and young child feeding products. The regulations were revised in 2004 to create more structured guidelines and include HIV/AIDS-related feeding recommendations. |
|  | Classification | Direct Health Sector Nutrition Intervention |
| Local Government Act (1997) | Description | The Local Government Act enacted the decentralization of the federal government to devolve functions, powers, and services to local governments. The act provided decentralization at all levels of local governments to ensure local populations are able to participate in decision-making. |
|  | Classification | Indirect Other Sectoral Strategy |
| Universal Primary Education (UPE) (1997-2007) | Description | UPE in Uganda was introduced in 1997 by eliminating tuition fees and Parents and Teachers Association (PTA) fees for public primary education. |
|  | Classification | Indirect Other Sectoral Strategy |
| National Health Policy (1999-2009) | Description | The overall objective of the first National Health Policy was to reduce mortality, morbidity, and fertility of all Ugandans. The policy also sought to ensure access to the Uganda National Minimum Health Care Package (UNMHCP), which includes interventions that address the major causes of the burden of disease. |
|  | Classification | Direct Health Sector Nutrition Intervention |
| National Guidelines on Planning and Implementation of Vitamin A Supplementation (2001) | Description | The aim of the National Guidelines on the Planning and Implementation of Vitamin A Supplementation were to improve the coverage of vitamin A supplementation in children 6-59 months of age to 95% by the end of 2005. The guidelines also provided recommendations to start vitamin A supplementation among non-breastfed infants under 6 months of age and postpartum women within eight weeks post-delivery. |
|  | Classification | Direct Health Sector Nutrition Intervention |
| Uganda Anemia Policy (2001) | Description | The Uganda Anemia Policy was enacted in 2001 with the aim of improving the management, early detection, and treatment of anemia. The policy included various intervention packages for pregnant women, adolescent girls, and preschool and school-aged children. |
|  | Classification | Very important to stunting decline |
| Uganda National Food and Nutrition Policy (NFNP) (2003) | Description | The aim of the Uganda National Food and Nutrition Policy is to ensure food security and improved nutrition for the Ugandan population. The policy covered twelve priority areas and nine nutrition-specific objectives. |
|  | Classification | Direct Health Sector Nutrition Intervention |
| Child Days Plus Strategy (2004) | Description | The Child Days Plus Strategy has been implemented biannually for 2 months each year to provide preventive health services at health facilities, community outreach sites, and primary schools. During these periods of accelerated action, health workers provide routine and catch-up immunization, vitamin A supplementation for all children under-5, deworming for children 1 to 14 years, tetanus immunization for women of reproductive age, and education on healthy practices such as breastfeeding, hygiene, and malaria. |
|  | Classification | Indirect Health Sector Strategy |
| Education Sector Strategic Plan (ESSP) (2004-2015) | Description | The ESSP aimed to create an education system relevant to Uganda’s national development goals, assist students in achieving education goals, and establish an efficient and effective education sector. |
|  | Classification | Indirect Other Sectoral Strategy |
| Food Fortification Guidelines (2005) | Description | The Food Fortification Guidelines encourage and promote the fortification of staple foods with one or more micronutrients by means of a fortificants or fortification mix. The guidelines also ban the manufacturing, import, and vending of fortified foods that are not in accordance with national standards. |
|  | Classification | Direct Other Sectoral Strategy |
| Health Sector Strategic Plan II (2005-2010) | Description | The Second Health Sector Strategic Plan focused on four key intervention areas: (1) health promotion, disease prevention, and community health initiatives; (2) maternal and child health; (3) prevention and control of communicable diseases; and (4) prevention and controls of NCDs. |
|  | Classification | Direct Health Sector Nutrition Intervention |
| Uganda Food and Nutrition Strategy and Investment Plan (UFNSIP) (2005) | Description | The primary purpose of the UFNSIP was to establish a strategic plan to implement the Food and Nutrition Policy by improving food security, nutrition, health, and well-being of the Ugandan population. |
|  | Classification | Direct Health Sector Nutrition Intervention |
| Employment Act (2006) | Description | The 2006 Employment Act mandated the provision of parental leave in Uganda. According to the Act, women are entitled to 60 paid days of maternity leave at full salary and job guarantee when returning to work. Men are entitled to 4 paid days of paternity leave after the delivery or miscarriage of a child and job guarantee upon return to work. |
|  | Classification | Indirect Other Sectoral Strategy |
| Pro-Poor Strategy for the Water and Sanitation Sector (2006) | Description | The Pro-Poor Strategy for the Water and Sanitation Sector was implemented to provide the poor with access to water. The primary interventions of this strategy were to: (1) allow communities to exempt or reduce water supply costs for the poor; (2) target funding to underserved areas; and (3) promote self-supply and rainwater harvesting. |
|  | Classification | Indirect Other Sectoral Strategy |
| U.S. President’s Malaria Initiative (PMI) (2006) | Description | The USAID-funded U.S. President’s Malaria Initiative (PMI) was launched in 2005 with the goal of reducing malaria-related mortality by 50% in 15 high-burden countries in sub-Saharan Africa. The initiative aimed to meet this goal through the rapid scale-up of four malaria prevention and treatment measures: (1) insecticide-treated mosquito nets (ITNs); (2) indoor residual spraying (IRS); accurate diagnosis and prompt treatment with artemisinin-based combination therapies (ACTs); and (4) intermittent preventive treatment of pregnant women (IPTp). |
|  | Classification | Indirect Health Sector Strategy |
| National Food Safety Strategic Plan (NFSSP) (2007-2016) | Description | The primary objectives of the NFSSP were to establish the National Food Safety Control System, ensure local manufactured and imported food met national standards, provide institutional framework and capacity, coordinate food safety-related activities, and reduce the burden of foodborne illnesses in the country. |
|  | Classification | Indirect Other Sectoral Strategy |
| Strengthening the National Food Fortification Programme (2007-2012) | Description | The Strengthening the National Food Fortification Programme was created in response to the high levels of micronutrient deficiency, specifically iron deficiency anemia, in Uganda. The programme sought to fortify vegetable oil, wheat, and maize flour by the end of 2012. |
|  | Classification | Direct Other Sectoral Strategy |
| Universal Secondary Education (USE) (2007) | Description | In 2007, Uganda became the first country in Sub-Saharan Africa to introduce USE. Students receive free secondary education in public schools and participating private schools, if they receive certain grades in each of the four primary school exit exams. The government pays the schools an annual grant of up to 141,000 UGX per student, while parents are still responsible for the costs of students’ uniform, stationary, and meals. |
|  | Classification | Indirect Other Sectoral Strategy |
| National Gender Policy (2007) | Description | The National Gender Policy was passed to develop: (1) interventions that respond to the diverse needs of women and men; (2) rudimentary technologies for poor women and men; and (3) incentive frameworks to improve the earning potential of poor women and men for improved productivity and output. |
|  | Classification | Indirect Other Sectoral Strategy |
| National Population Policy for Social Transformation and Sustainable Development (2008) | Description | The purpose of the National Population Policy for Social Transformation and Sustainable Development is to improve the quality of life and living standards for future demographics. This policy specifically focuses on children, youth, women, elderly, and persons with disabilities. |
|  | Classification | Indirect Health Sector Strategy |
| Child Survival Strategy (CSS) (2008-2015) | Description | The Child Survival Strategy was created to provide policymakers with a clear direction of priority actions required to reduce under-5 mortality in Uganda. The goal of this strategy was to reduce the under-5 mortality rate from 137 per 1,000 live births to 56 per 1,000 live births by 2015. The strategy proposed a priority package of cost-effective child survival strategies, which covered maternal and newborn healthcare, appropriate treatment of major childhood diseases, vaccination against preventable diseases, nutrition interventions, malaria prevention and treatment, HIV prevention and treatment, and water and sanitation interventions. |
|  | Classification | Direct Health Sector Nutrition Intervention |
| Zinc Supplementation Guidelines (2008) | Description | The goal of the Zinc Supplementation Guidelines is to reduce morbidity and mortality caused by diarrheal diseases. As a result, the strategy recommends zinc supplementation for all children suffering from diarrhea. Further implementation and evaluation information is unavailable (69). |
|  | Classification | Direct Health Sector Nutrition Intervention |
| National Nutrition Operational Framework for Nutrition in the National Child Survival Strategy (2009) | Description | This framework comprises of eight thematic objectives that cover the nutrition component of the Child Survival Strategy. |
|  | Classification | Direct Health Sector Nutrition Intervention |
| Nutrition in the Context of HIV and Tuberculosis Strategic Plan (2009-2014) | Description | This strategy created a national framework for implementing food and nutrition interventions in the context of HIV tuberculosis (TB) programs. |
|  | Classification | Direct Health Sector Nutrition Intervention |
| Reproductive Health Commodity Security Strategic Plan (2009-2014) | Description | The purpose of the Reproductive Health Commodity Security Strategic Plan was to ensure that every person in Uganda has access to quality contraceptives and other reproductive health commodities in order to contribute to accelerating the reduction of maternal and neonatal morbidity and mortality in the country. |
|  | Classification | Indirect Health Sector Strategy |
| Uganda Policy Guidelines on Infant and Young Child Feeding (2009) | Description | The aim of the Uganda Policy Guidelines on Infant and Young Child Feeding is to provide a framework for ensuring the survival of infants and young children by strengthening the care and support of IYCF services to their parents and caretakers. The guidelines include IYCF recommendations under “normal” circumstances, as well as the feeding of infants and young children exposed to HIV or in other difficult situations. |
|  | Classification | Direct Health Sector Nutrition Intervention |
| Agricultural Development Strategic Investment Plan (2010-2015) | Description | The purpose of the Agricultural Development Strategic Investment Plan was to invest in the agricultural sector to ensure efficient and effective provision of agricultural public goods, services, and support. |
|  | Classification | Indirect Other Sectoral Strategy |
| Integrated Management of Acute Malnutrition (IMAM) Guidelines (2010) | Description | The IMAM Guidelines focus on reducing malnutrition by improving the identification, treatment, and management of acute malnutrition. |
|  | Classification | Direct Health Sector Nutrition Intervention |
| National Malaria Control Strategic Plan (2010-2015) | Description | The goal of the National Malaria Control Strategic Plan was to rapidly scale-up the coverage of effective malaria prevention and treatment interventions to reduce the prevalence of malaria in Uganda, and thereby improve health outcomes. |
|  | Classification | Indirect Health Sector Strategy |
| Uganda National Malaria Control Policy (2010-2015) | Description | The Uganda National Malaria Control Policy aimed to provide all malaria-control stakeholders and partners with a single policy framework for malaria control in the country. The policy complemented the National Malaria Control Strategic Plan. It had 10 key areas of focus: malaria case management; malaria diagnostics; treatment; malaria in pregnancy; malaria vector control; advocacy and social mobilization; epidemic preparedness and response; health systems strengthening; monitoring and evaluation; and research. |
|  | Classification | Indirect Health Sector Strategy |
| Uganda Nutrition Action Plan (UNAP) (2011-2016) | Description | The goal of the Uganda Nutrition Action Plan was to reduce the levels of malnutrition among women of reproductive age, infants, and young children through 2016. |
|  | Classification | Direct Health Sector Nutrition Intervention |
| Scaling Up Nutrition (SUN) (2011) | Description | Uganda joined SUN in 2011, an international, multi-sectoral collaboration that aims to end all forms of malnutrition by 2030. It has a number of global targets including a reduction in stunting, wasting, and low birth weight, a reduction in anemia among reproductive age women, an increase in breastfeeding for the first 6 months, and no increases in overweight, obesity, and diabetes. |
|  | Classification | Direct Health Sector Nutrition Intervention |
| Sustainable Comprehensiveness Responses for Vulnerable Children and their Families (SCORE) (2011-2018) | Description | The aim of the SCORE program was to decrease the vulnerability of moderately and critically vulnerable children and their households through multi-sectoral, family-centered interventions that targeted various household members. Increased savings, market skills, apprenticeship, and farming/horticulture were classified as economic interventions, while life skills for youth, parenting skills trainings for parents, nutrition education/cooking demonstrations, and community dialogues on child protection, hygiene, and health issues were categorized under “other” interventions. |
|  | Classification | Indirect Other Sectoral Strategy |
| United Nations Renewed Efforts Against Child Hunger and Undernutrition (REACH) Initiative (2011-present) | Description | The REACH Initiative, which supports participating countries in strengthening the governance and management of nutrition programs, was expanded to Uganda in 2011. The initiative was launched in 2008 in partnership with WFP, FAO, WHO, and UNICEF. |
|  | Classification | Direct Health Sector Nutrition Intervention |
| World Food Program’s Safety Nets Policy (2012) | Description | The Safety Nets Policy was implemented in Uganda by the World Food Program in 2012. Main activities of this policy include school feeding, nutrition, food security, livelihoods, and smallholders. Further policy information is unavailable. |
|  | Classification | Direct Health Sector Nutrition Intervention |
| National Agriculture Policy (2013) | Description | The National Agriculture Policy was implemented in 2013 to ensure food and nutrition security and improve household incomes.. |
|  | Classification | Indirect Other Sectoral Strategy |
| Reproductive, Maternal, Newborn, and Child Health Sharpened Plan for Uganda (2013) | Description | The goal of the Reproductive, Maternal, Newborn, and Child Health Sharpened Plan is to end preventable maternal and child deaths in Uganda. The plan aims to: (1) accelerate greater coverage in high-burden districts and populations; (2) expand coverage of high impact interventions; (3) harness non-health sector interventions that impact maternal, newborn, and child vulnerability and deaths; and (4) develop and sustain collective action and mutual accountability for ending preventable maternal, newborn, and child deaths. |
|  | Classification | Indirect Health Sector Strategy |
| Uganda National Expanded Programme on Immunization (UNEPI) (2012-2016) | Description | The UNEPI was implemented from 2012-2016 to ensure that every child and high-risk group was fully vaccinated against targeted diseases according to recommended strategies. The program ran routine immunization services at health facilities, through outreach services, and national immunization days. |
|  | Classification | Indirect Health Sector Strategy |
| Food and Nutrition Technical Assistance (FANTA) III Project (2013-2018) | Description | The FANTA Project was a project funded by USAID to improve the health and well-being of underserved families and communities in developing countries by strengthening food security and the country’s nutrition landscape. |
|  | Classification | Direct Other Sectoral Strategy |
| Malaria Reduction Strategic Plan (2014-2020) | Description | The Malaria Reduction Strategic Plan was implemented in 2014 to provide quality malaria prevention and treatment services to the Ugandan population. By 2020, the plan aimed to: (1) reduce annual malaria deaths from the 2013 level to near zero; (2) reduce malaria morbidity to 30 cases per 1,000 population; and (3) reduce the malaria parasite prevalence to less than 7%. |
|  | Classification | Indirect Health Sectoral Strategy |
| Communication for Healthy Communities (CHC) (2014-2019) | Description | The CHC program was implemented in 2014 by USAID. The program was aimed at supporting the Government of Uganda in designing and implementing health communication interventions that contribute to the reduction in the high national rates of HIV infection, total fertility, maternal and child mortality, malnutrition, malaria, and TB. |
|  | Classification | Indirect Health Sector Strategy |
| National Malaria Control Program (2014-2020) | Description | The National Malaria Control Program guided malaria control efforts as outlined in the Malaria Reduction Strategic Plan. The overall goal of the plan was to reduce mortality due to malaria by 80% of the 2010 levels and reduce morbidity due to malaria by 75% of the 2010 levels. |
|  | Classification | Indirect Health Sector Strategy |
| Strengthening Human Resources for Health (SHRH) Activity (2014-2019) | Description | The SHRH Activity was a USAID-funded project that worked with the Government of Uganda to address and manage the national health workforce for improved health, HIV/AIDS and nutrition services, and better health outcomes. The objectives were approached using multi-sectoral collaboration between district governments, training institutions, health professional councils, IntraHealth, Ministries of Health, Education, and Public Service. |
|  | Classification | Indirect Health Sector Strategy |
| The National Integrated Early Childhood Development (NIECD) Policy Action Plan of Uganda (2016-2021) | Description | The NIECD Policy of Uganda was enacted in 2016 to provide direction and guidance to all sectors for quality, inclusive, coordinated, and well-funded ECD services and programs. |
|  | Classification | Indirect Other Sectoral Strategy |
| *SUBNATIONAL-LEVEL POLICIES/PROGRAMS* | | |
| Northern Uganda Social Action Fund (NUSAF) 1 Project (2002-2009) | Description | The first NUSAF I Project ran from 2002 to 2009, with funding from the World Bank. It aimed to provide social protection and funds to vulnerable groups living in 18 districts of Northern Uganda. |
|  | Classification | Indirect Other Sectoral Strategy |
| Uganda Programme for Human and Holistic Development (UPHOLD) (2002-2008) | Description | The USAID-funded UPHOLD was implemented by John Snow Inc. The program began in 20 districts in 2003 and expanded its coverage to 34 districts by 2006. UPHOLD targeted districts in West, Central, East, South West, North East, and North Uganda to: (1) improve educational status; (2) reduce the spread of HIV/AIDS and sexually transmitted infections; (3) decrease maternal and child mortality; and (4) stabilize population growth. |
|  | Classification | Direct Health Sector Nutrition Intervention |
| World Food Program Food for Assets (FFA) Programme (2002-2014) | Description | The WFP-funded Food for Assets (FFA) program is currently being implemented in 55 countries. It was implemented from 2002-2014 in the Acholi, West Nile, Karamoja, Teso, and Lango regions of Uganda. FFA addressed food security through cash, vouchers, and food transfers, while also promoting the strengthening of assets that will improve long-term security and resilience. |
|  | Classification | Indirect Other Sectoral Strategy |
| Gender Informed Nutrition and Agriculture (GINA) Project (2005-2007) | Description | The GINA project was funded by USAID and piloted in Uganda, Mozambique, and Nigeria. The project was implemented in the Kabale, Kanungu, and Rukungiri districts of Uganda for 24 months, between 2005 and 2007. |
|  | Classification | Direct Other Sectoral Strategy |
| Stop Malaria Project (SMP) Uganda (2008-2015) | Description | The SMP was a USAID-funded program that aimed to help the Ugandan government reach the Roll Back Malaria goal of reducing malaria-related morbidity and mortality by 70% by 2015 and contribute to the achievement of the Millennium Development Goals (MDGs). SMP had 34 project supported districts in the Mid-Western, Central, and Teso regions. |
|  | Classification | Indirect Health Sector Strategy |
| NUSAF 2 (2009-2016) | Description | The second NUSAF project was implemented from 2009 to 2016 across 55 districts in Northern Uganda. The objective of this project was to improve income earning opportunities and access to better basic socioeconomic services for beneficiary households in the region. |
|  | Classification | Indirect Other Sectoral Strategy |
| Agricultural Livelihoods Recovery Programme (ALREP) (2010-2014) | Description | ALREP was a direct income support program implemented by the Government of Uganda in the subregions of Lango, Acholi, and Teso in Northern Uganda. The objective of the program was to ensure food security and increased household income in the war-affected population of Northern Uganda through productive and profitable agricultural and agri-business activities. |
|  | Classification | Indirect Other Sectoral Strategy |
| Karamoja Productive Assets Programme (2010) | Description | The Karamoja Productive Assets Programme was a joint initiative by WFP, FAO, and the Government of Uganda to improve the food security in drought-affected areas of Karamoja through social safety nets. |
|  | Classification | Indirect Other Sectoral Strategy |
| Social Assistance Grants for Empowerment (SAGE) Programme (2010) | Description | The SAGE program was a cash transfer scheme initiated by the Ministry of Gender, Labour, and Social Development targeting the poorest 10% of households in 15 districts of the country, which were mostly concentrated in Northern Uganda. |
|  | Classification | Indirect Other Sectoral Strategy |
| Karamoja Integrated Development Programme (KIDP) (2011-2015) | Description | The KIDP was implemented from 2011 to 2015 in the Karamoja subregion of Uganda and had seven multisectoral objectives. |
|  | Classification | Indirect Other Sectoral Strategy |
| Advocacy for Better Health (ABH) (2014-2018) | Description | The USAID-funded ABH project aimed to improve the availability, accessibility, and quality of health and social services in 35 target districts across Uganda. ABH supported initiatives that were related to HIV/AIDS and TB, malaria, nutrition, MNCH, reproductive health, and family planning, and targeted women, children, youth, and marginalized and at-risk populations. |
|  | Classification | Indirect Health Sector Strategy |
| Regional Health Integration to Enhance Services in Southwest Uganda (RHITES-SW) (2015-2020) | Description | The aim of the USAID-funded RHITES-SW program was to increase the adoption of healthy behaviors and positive child development practices. It provided services in 771 health facilities across 16 districts in the Southwest region: Mbarara, Isingiro, Buhweju, Ibanda, Kiruhura, Sheema, Bushenyi, Rubirizi, Ntungamo, Mitooma, Kabale, Rubanda, Kisoro, Rukiga, Kanungu, and Rukungiri. |
|  | Classification | Indirect Health Sector Strategy |
| HarvestPlus Meals for Nutrition in Uganda (MENU) (2016-2021) | Description | The purpose of the MENU project was to reduce micronutrient malnutrition and improve dietary intakes of vitamin A and iron in 4 districts (Lira and Dokolo in Northern Uganda, and Mpigi and Butambala in Central Uganda) of Uganda by 2021. |
|  | Classification | Direct Health Sector Nutrition Intervention |
| Malaria Action Program for Districts (2016-2021) | Description | The USAID-led Malaria Action Program for Districts was implemented in 43 districts across the Central, Western, and West Nile regions where malaria was most prevalent. |
|  | Classification | Indirect Health Sector Strategy |
| NUSAF 3 (2016-2020) | Description | The third NUSAF project aimed to provide effective income support to and build the resilience of poor and vulnerable households across 62 districts of Northern Uganda. |
|  | Classification | Indirect Other Sectoral Strategy |
| Regional Health Integration to Enhance Services in East Central Uganda (RHITES-EC) (2016-2021) | Description | The USAID-funded RHITES-EC supported the federal Ministry of Health to improve regional health outcomes in 12 districts of East Central Uganda by increasing the utilization of high-quality healthcare services. |
|  | Classification | Indirect Health Sector Strategy |
| Regional Health Integration to Enhance Services in Eastern Uganda (RHITES-E) (2017-2022) | Description | The RHITES-E project is a USAID-funded project that targets 23 districts in Eastern Uganda and Karamoja. RHITES-E aims to generate stronger demand for HIV/AIDS, TB, MNCH, reproductive health, nutrition, and malaria services by: (1) improving data use; (2) integrating health services; (3) introducing digital health solutions; and (4) improving the quality of services provided. |
|  | Classification | Indirect Health Sector Strategy |
